# Protective effects of *Marinobacter nauticus* strain GH3 exopolysaccharide on the *Oreochromis niloticus* model for Alzheimer’s disease

**DOI:** 10.1038/s41598-024-78036-8

**Published:** 2024-11-11

**Authors:** Ghada Abdel-Razik, Mohamad Abdelrazik, Alaa Rashad, Wagdy K. B. Khalil, Fagr Kh. Abdel-Gawad, Ahmed A. Hamed, Mohamed E. El Awady

**Affiliations:** 1 Botany and Microbiology Department, Faculty of Science, Suez Canal University, Ismailia, 41522 Egypt; 2 Center of Excellence for Research and Applied Studies on Climate Change and Sustainable Development, National Research Centre (NRC), 33 El Bohouth St. Dokki, Giza, 12622 Egypt; 3 Cell Biology Department, Centre of Excellence for Advanced Science (CEAS), National Research Centre (NRC), 33 El Bohouth St. Dokki, Giza, 12622 Egypt; 4 Water Pollution Research Department, Centre of Excellence for Advanced Science (CEAS), National Research Centre (NRC), 33 El Bohouth St. Dokki, Giza, 12622 Egypt; 5 National Biotechnology Network of Expertise (NBNE), Academy of Scientific Research and Technology (ASRT), Cairo, 11516 Egypt; 6 Microbial Chemistry Department, National Research Centre, El-Buhouth St. 33, Dokki-Cairo, Giza, 12622 Egypt; 7 Microbial Biotechnology Department, National Research Centre, El-Buhouth St. 33, Dokki- Cairo 12622, Giza, Egypt

**Keywords:** Exopolysaccharide, Alzheimer’s disease, Neurodegenerative disorders, Immunomodulatory, Antioxidant, *Oreochromis niloticus*, Carbohydrates, Drug development, Applied microbiology, Bacteria, Clinical microbiology

## Abstract

*Marinobacter nauticus* strain GH3 was isolated from the Red Sea, Sharm Elshiekh, and classified according to cultural attributes, biochemical properties, and the analysis of genetic relationships using 16 S rRNA sequences. A substantial proportion of exopolysaccharides (EPS) in GH3-EPS contained a sulfate content of 25.4%, uronic acid (12.18%), and N-acetylglucosamine (13.6%). The composition of monosaccharides in this fraction consists of glucose, glucoronic acid, arabinose, and xylose by 2:4:3:3, respectively. SEM showed a flower-like shape with white bundles on the GH3-EPS surface. GH3-EPS enhancement of the RAW264.7 macrophage line RAW 264.7 ATTC number J774 cell proliferation via MTT assay for cell viability. GH3-EPS had a high stimulation effect on releasing TNF-alpha and IL-10. Followed by its effect against cyclooxygenase (COX-2) and lipoxygenase (LOX), with IC_50_s of 14.74 and 19.4 µg/ml, respectively. Antioxidant activity was evaluated for GPx-4, GSS, and MDA with highly significant results, and for DPPH, ABTS, and iron chelating with IC_50_ (43.51, 31.27, and 84.96 µg/ml, respectively). AChE was inhibited by a mean of 52.92 ± 4.54 and 68.22 ± 5.64 µg/ml. In a fish animal model, GH3-EPS demonstrated a positive treatment effect for AD, supporting biochemical studies, histopathology for some brain parts, and toxicity.

## Introduction

Marine bacteria have a significant impact on the biogeochemical processes in the oceans. They produce a wide range of biomolecules found in both water’s different heights and sediments. These biomolecules can interact with other organisms or non-living surfaces either independently or in association with them^1^. Naturally, these biomolecules exhibit chemical heterogeneity and possess a wide range of biological functions such as biofilm formation, competition, feeding, and more. Their chemical makeup can consist of proteins, carbohydrates, lipids, or a combination of these. Certain molecules, such as enzymes, biosurfactants, and exopolysaccharide (EPS), possess a polymer structure^2^. Microorganisms release soluble or insoluble polymers known as EPSs. Moreover, bacteria are regarded as relatively consistent structures among all polysaccharide producers and are tightly controlled, while exopolysaccharide (EPS) structures derived from plant and animal sources are affected by climatic, environmental, and dietary conditions^3^.

Extracellular polymeric compounds are intricate mixtures of microbial biopolymers with high molecular weight that are secreted as byproducts. Essentially, they consist of proteins, polysaccharides, uronic acids, humic compounds, lipids, and other components. Extracellular polysaccharides, often known as exopolysaccharides, are used for ion sequestration (EPS). The composition of this substance consists mostly of intricate, large organic macromolecules with a high molecular weight, such as polysaccharide. There are also trace levels of protein and uronic acid in it^4,5^. Capsular polysaccharide (CPS) or a slimy coating on microbial surfaces are two possible locations for the synthetic polymers^6^. They are generated as a response to environmental stress in order to defend against dehydration or harmful compounds such as nickel (Ni). Additionally, they function as a source of carbon and energy^7^.

Nickel (Ni) is a potential neurotoxic pollutant, and the nervous system is one of the systems affected by Ni toxicity^8^. Studies have indicated how nickel chloride can cause mutagenic, cancerous, immunotoxic, and DNA damage^9,10^. The various media of nickel exposure (Ni) compounds have generated significant public health worries, as they have been demonstrated to have harmful effects on biological organs, including the brain and Alzheimer’s disease (AD)^11^. Of all dementias, Alzheimer’s disease (AD) occurs most frequently. The primary clinical manifestation of Alzheimer’s disease (AD) is a gradual deterioration of memory. Nevertheless, AD can also be characterized by the impairment of speech and motor skills, feelings of sadness, false beliefs, visual or auditory perceptions that are not based in reality, and hostile conduct^12^. Although there are noticeable variations in behavioral defects, accurately diagnosing someone with AD in the early stages of the disease is challenging^13^. There is significant evidence that AD patients exhibit substantial neuronal degeneration in multiple brain regions^14^.

*Oreochromis niloticus*, often known as Nile tilapia, is highly favored by fish growers due to its exceptional growth performance in many aquatic environments^15^. The production of Nile tilapia (*Oreochromis niloticus*) has had significant and rapid development in recent years. In 2015, global production reached about 5.6 million metric tons, making it the second most cultivated fish species worldwide^16^. Nile tilapia is considered a primary species with excellent potential for use in Egypt’s freshwater aquaculture. This is owing to the fast rate of growth and its remarkable capacity to adapt to various environmental circumstances, high-quality meat, strong market demand, and well-established breeding practices^17,18^. However, the process of fish intensification is considered to cause physical suffering, which might lead to immune suppression and increased susceptibility to diseases in fish. Therefore, to study the effects of different amounts of toxic contaminants on the brain, Nile tilapias were used as appropriate aquatic models.

The present work is carried out with the aim of isolating and identifying exopolysaccharide-producing bacteria. Additionally, studying the histopathological effects of GH3-EPS on the brain of *O. niloticus* fish after exposure to the LC_50_ of nickel chloride.

## Materials and methods

### Isolation and identification of an exopolysaccharide-producing bacterial strain

Samples were gathered from Sharm El Sheikh Sea water and sediment, Egypt [27° 5456.95” N, 34° 1947.82” E]. The serial dilution plating technique was applied to seawater yeast extract peptone (SYEP) for bacterial isolation^19^. Plates were then incubated at 28 ± 2 °C for 24 h with a PH of 8.0. Bacterial colonies were picked up at random from each plate and streaked onto SYEP agar to obtain pure cultures, which were then used for screening. The isolate that generated the highest EPSs and had antioxidant activity was identified using the sequencing of the 16 S rRNA gene^20^. A universal primer was obtained from Integrated DNA Technologies of India for the purpose of amplifying the sequencing of the 16 S rDNA gene. The study used the following primer sequences: Reverse 27 F (**5**′-AGAGTTTGATCCTGGCTCAG-**3**′) and forward 1492R (**5**′-TACGGYTACCTTGTTACGACTT-**3**′)^21^. The 16 S rRNA sequences were aligned and compared using the BLAST-n tool, which may be found at http://www.ncbi.nlm.nih.gov/BLAST. Subsequently, we submitted the entirety of the sequences we had gathered to GenBank in order to obtain accession numbers. The Bio-Edit tool was used to align the 16 S rRNA sequences, excluding regions containing ambiguous nucleotides. The pyogenesis tree was constructed using the neighbor-joining statistical method available in the Mega X software tool, which can be obtained from https://www.megasoftware.net/dload_win_gui.

### EPS extraction and partial purification

The strain with high potential was selected for the generation of EPS. The fermentation medium consisted of broth containing sucrose at a concentration of 20 g/L, yeast extract at a concentration of 2 g/L, and peptone at a concentration of 4 g/L. This broth was dissolved in 750 ml of seawater and then diluted to a final volume of 1 L in order to exclude bacterial organisms. Centrifugation was used to separate the samples while they were frozen at 4 °C for 30 min after the specimen was taken. The solution was heated to 4 ºC in an incubator after adding 10% trichloroacetic acid (TCA). After that, the protein was extracted from the solution by centrifuging it at 5000 rpm for 20 min. A NaOH solution was used to neutralize the pH of the supernatant to 7^22^. The bacterial mass was obtained through centrifugation following the addition of 4 L of extremely cold ethanol to the liquid portion. The residual material was dissolved again within deionized H_2_O, followed by a 72-hour dialysis treatment using deionized water. Absolute cold ethanol in volumes of 1, 2, 3, and 4 L was used for fractional precipitation of the dialyzed solution, respectively. The UV absorption spectra were obtained in the wavelength range of 200 to 800 nm to determine the presence of proteins and nucleic acids^22^. For the determination of cell dry weight and the production of exopolysaccharides in the isolate, bacteria were grown for three days. Then, it was harvested and dried at 80 ^º^C to a constant weight. The purification of the EPS fraction was concentrated and adsorbed onto DEAE-cellulose. The non-absorbed EPS was washed using distilled water, while the absorbed EPS was separated using a stepwise elution technique using NaCl concentrations between 0.2 M and 1.0 M to separate the absorbed EPS. With a flow rate of 1 ml/min, phenol-sulfuric acid was used to test^23^. The active fractions were combined, dialyzed using deionized water, and then precipitated with ethanol after concentration.

### GH3-EPS characterization

The determination of total sugars was performed using the phenol-H_2_SO_4_ technique^23^, using glucose as a standard for comparison. In order to measure uronic acid, use the m-hydroxybiphenyl method^24^. The investigation used glucuronic acid as the reference standard. Sulfate analysis was conducted after hydrolysis using an 85% concentration of formic acid at a temperature of 100 °C for a duration of 5 min. The turbidimetric method^25^ was employed for this objective, using sodium sulfate as the reference substance. Analyzing the monosaccharide content of GH3-EPS was conducted using the Shimadzu Shim-Pack SCR-101 N column in high-performance liquid chromatography at 7.9 mm × 30 cm. The mobile phase used in the experiment was deionized water, and it was flowing at a rate of 0.5 mL per minute at a temperature of °C^26^. The process of sugar identification involved the comparison of the sample with pure sugar samples. Examined with the use of a 500-IR FTIR spectrophotometer made by the Bucker Company in Ettlingen, Germany, the Fourier transform infrared (FT-IR) spectra of GH3-EPS were examined. The spectrum was obtained in the wavenumber range of 4000–400 cm1. The purified GH3-EPS was ground with spectroscopic-grade KBr powder and then compressed into pellets to facilitate (FT-IR) measurements^27^.

### Evaluation of systemic acute toxicity effects of GH3-EPS

#### Animal

A group of ten male albino rats, each weighing between 100 and 150 g, were provided by the Animal House of the National Research Centre (Cairo, Egypt). Then, housed in polycarbonate cages with a maximum capacity of four rats per cage. The subjects were situated in a regulated setting, upheld by a 12-hour cycle of light and darkness, and kept at a temperature of 24 ± 2 ºC using an air conditioning system. The humidity levels were maintained between 50 and 70%. All care and procedures used in the experiments were in accordance with the ARRIVE guidelines and approved by the Ethics Committee from the Institutional Animal Ethics Committee of Suez Canal University, Egypt [No. REC67/2022]. All methods were performed in accordance with the relevant guidelines and regulations.

The lethal toxicity (LD_50_) of the GH3-EPS was determined in male rats using the Meier technique. The dose levels of 500 mg were arranged in a regular progression, increasing in concentration. All injections were administered intraperitoneally. The mortality and survival rates of the treated animals were documented 24 h after the injection^28^. This study employed ten white male rats to assess the impact of GH3-EPS on the liver, brain, kidney, and heart, which are the main organs of interest. Rats were administered GH3-EPS orally at varying dosages based on their body weight. The administration was done once daily for 14 days. At the end of the study period (specifically, on Day 14).

#### Preparing a blood sample and biochemical parameters

At the end of the study period, the rats were administered anesthesia with ketamine and xylazine at a dosage of ketamine 80–100 mg/kg and xylazine 10–12.5 mg/kg^29^. Then, all experimental rats were euthanized by carotid exsanguination just after loss of consciousness. Blood samples were obtained from the retroorbital venous plexus using capillary tubes that were treated with heparin. The blood samples were subjected to centrifugation at a speed of 3000 rpm for a duration of 10 min. The sera were kept at a temperature of -20 °C until they were analyzed to determine biochemical characteristics. An automated biochemistry analyzer was used to ascertain the hematological parameters as well as the values of ALT, AST, GLU, ALP, TC, TG, and UN.

#### Histological evaluation

The heart, liver, kidney, and brain samples from all experiments were fixed with neutral buffered formalin at a 10% concentration. Prior to being embedded in paraffin wax, the fixed samples underwent dehydration in an escalating series of ethanol. They were then cleaned with xylene. A microtome was used to prepare sections with a thickness of 5 μm. The sections were then stained with hematoxylin and eosin (H and E) and examined under a light microscope.

### Assays for the immune-related index of GH3-EPS

#### Culturing macrophages

Macrophage cell lines RAW 264.7 ATTC obtained from the microbiology department, faculty of medicine for girls, Al-Azhar University in Cairo, Egypt, were maintained in DMEM that was enhanced with 100 U/mL penicillin, 100 µg/mL streptomycin, and 10% fetal bovine serum. The cells were kept in a 37 °C humidified atmosphere with 5% CO_2_ for incubation in a 25 cm^2^ cell culture flask. Every three to four days, the cells were subcultured to be used in all experiments^30^.

#### Cell viability assay

The cell viability was assessed using the 3-(4,5-dimethylthiazol-2-yl)-2,5-diphenyltetrazolium bromide (MTT) test. Different concentrations of GH3-EPS (31.25–2500 µg/mL)^31^. Briefly, macrophage cell line RAW 264.7. was incubated in a 96-well plate and maintained in the growth medium for 24 h at 37 °C. Following the formation of the cell monolayer, the tested sample was diluted in RPMI medium with 2% serum twice. The plate was examined for signs of toxicity, and an MTT solution was prepared. The MTT was then incubated for 1–5 h, and the media was discarded. The optical density was then measured at 560 nm and 620 nm.

#### Cytokines expression by GH3-EPS

Monitoring cytokine concentrations in the supernatants of medium from the macrophage cell line described above in the MTT assay allowed us to identify GH3-EPS’s immunomodulatory effects. They were determined by the Human Interleukin 10, IL-10 ELISA Kit (E0102Hu) and the Human Tumor Necrosis Factor Alpha (ELISA) Kit (E0082Hu) according to the manufacturer’s guidelines. All kits employ the quantitative sandwich enzyme immunoassay technique. These findings were presented as cytokine concentrations in the culture medium (Pg/ml)^32,33^.

### Antioxidant-related index assays for GH3-EPS

#### Enzymatic test

In order to identify GH3-EPS’s antioxidant activity, glutathione synthetase (GSS) and glutathione peroxidase-4 (GPx-4) were determined using the macrophage cell culture supernatant as described before using Rat Glutathione Peroxidase 4; GPX-4 ELISA Kit (E1787Ra) and Human Glutathione Synthetas; GSS ELISA Kit (E5347Hu), respectively, according to the manufacturer’s instructions^34–36^.

#### Malondialdehyde assay of GH3-EPS

Amounts of lipid peroxidation (LPO) were measured by analyzing the amount of malondialdehyde (MDA) through its reaction with thiobarbituric acid (TBA) at a wavelength of 532 nm using a previously established method^37^. The method relied on spectrophotometrically measuring the colored complex produced by the reaction between TBA and MDA.

#### DPPH (2,2-diphenyl-1-picrylhydrazy) radical scavenging assay of GH3-EPS

The DPPH assay was conducted according to the methodology described by Brand-William et al.^38^. In summary, 200 µL of a solution containing DPPH and ethanol as its concentration (0.1 mM) were combined with 100 µL of a GH3-EPS solution containing varying quantities ranging from 50 to 400 µg/mL. The mixture was left to incubate for half an hour at 37 °C. At a specific wavelength (517 nm), the absorbance of the test mixture was recorded. The sample solution was substituted with ethanol to serve as a blank control, and ascorbic acid was used as the reference material. We were able to measure the DPPH radical’s scavenging activity as:

Scavenging rate (%) = [(A _control_ − A _sample_) /A _control_] x 100.

#### The ABTS radical scavenging assay of GH3-EPS

The mixture of potassium persulfate (2.45 mM) and ABTS (7 mM) was incubated at 25 °C for 16 h^39^. The solution mixture was diluted with deionized water until it reached a specific absorbance value of 0.70 ± 0.02 at a wavelength of 734 nm. 100 µL of ABTS solution were supplemented with 50 µL of GH3-EPS solution, which had a concentration ranging from 50 to 400 µg/mL. Following a reaction during a 6-minute period at a temperature of 25 °C, the absorbance was measured using a wavelength of 734 nm. Subsequently, for the purpose of serving as a control, deionized water was used in place of the sample. The impact of ABTS scavenging was determined via calculation:

Scavenging rate (%) = [(A _control_ − A _sample_) /A _control_] x 100.

#### Fe2+ chelating of GH3-EPS

This Fe^2+^ test had been carried out according to the method described by^40^. In summary, a total of 220 µL of GH3-EPS solutions with concentrations varying from zero up to 1000 µg/mL had been combined with 5 µL of FeCl_2_ at a concentration of 2 mM. Subsequently, 10 µL of ferrozine at a concentration of 5 mM was added to the mixture. The resulting solution was thoroughly mixed and allowed to stand for 10 min at an average temperature of 25 °C. A measurement of absorbance was made at a wavelength of 562 nm. Ascorbic acid (Ac) was used as the sample, while deionized water was employed as the blank control. The chelating activity was determined through calculation:

Chelating rate (%) = [(A _control_ − A _sample_) /A _control_] x 100.

### The inflammatory intervention ability of GH3-EPS

The anti-inflammatory response of GH3-EPS and the reference substance (Ibuprofen) was assessed by inhibiting the LOX and COX-2 enzymes. The experiment was conducted, implementing the approach outlined throughout^41^. Applying this formula = (1 - As/Ac) x 100, the inhibitory activity (%) is calculated, whereas as indicates the absorbance while the test drug is present and Ac indicates the absorption of the control.

### Cholinergic intervention system of GH3-EPS on AD

The cholinesterase activity assay involved testing the inhibitory effect of different doses (50 and 150 µg/ml) of GH3-EPS on acetylcholine esterase^42^.

### Tilapia fish, *Oreochromis niloticus*, as an animal model for AD

#### Fish source, feed, and acclimation

Eighty Tilapia fish (*Oreochromis niloticus*) with an average body weight of 36 ± 3.2 g were retrieved from the Nubaria, Egypt, farm of the National Research Center. Tilapia fish have been administered lidocaine (CHNO) with a concentration of 5 mg/L during transit to reduce stress. The fish were transported for around two hours to a system of outdoor experiment enclosures known as mesocosms at the National Research Centre laboratory. They were placed in a fiberglass container with a water capacity of 1 m^3^, which was equipped with battery-powered aerators to provide oxygen. Under natural light, the fish were housed in a glass mesocosm (eight mesocosms total, 10 fish each). The mesocosms were exposed to natural light and filled with tap water that was not treated with chlorine. The temperature is 27.2 ± 1.8 °C, the pH level is between 7 and 8, and the concentration of dissolved oxygen is between 7 and 8 mg/L. A diet in pellet form was formulated using the methodology described by Abdel-Gawad et al.^43^.

#### Experimental design

Healthy Nile Tilapia were taken for the experiment and distributed into eight tanks (a–h), previously washed with 1% potassium permanganate to free the walls from microbial infection, if any. Following the procedure laid out by Wan et al.^44^, eighty fish were stocked into each of the eight tanks with a density of 10 fish per treatment. (a) The negative control group was fed only a fish pellet diet. (b & c & d) groups were only fed with a fish pellet diet in addition to different concentrations of GH3-EPS (low: 0.075; medium: 0.15; high: 0.3 gm) for toxicity evaluation. (e) The positive control group was fed only with a fish pellet diet in addition to adding NiCl_2_ regarding LC_50_ (0.1134 g/L)^45^. (f & g & h) groups were fed with a fish pellet diet in addition to different concentrations of GH3-EPS (low: 0.075; medium: 0.15; high: 0.3 gm), respectively, and exposed to NiCl_2_ LC_50_ (0.1134 g/L). During the 42-day feeding trial, the animals were fed twice a day at a ratio of 3% by body weight, and their food consumption was documented^44^. To keep the water quality high, about 25–30% of the water was changed every two days. Every day, syphoning at the bottom of the tanks was used to collect uneaten feed and excrement^44^. To make a nickel chloride stock solution (NiCl_2_ 6H2O), we dissolved an appropriate amount of NiCl_2_ 6H_2_O as Ni salt in distilled water. The LC_50_ was calculated regarding the ratio (1:10 of 3.6 mg NiCl_2_ per liter), respectively. This experiment followed the approval received from REC67/2022 reported from the botany and microbiology department, faculty of science, Suez Canal University. All methods were performed in accordance with the relevant guidelines and regulations.

#### Histopathological study

Randomly, three fish from every group were euthanized in 2-phenoxyethanol and dissected. The brains were eliminated and washed in saline solution, preserved in formalin, and dehydrated using a sequence of ethanol solutions. Subsequently, they were incorporated into a paraffin wax matrix. The blocks were sliced into sections using a microtome with a thickness of 5 μm. Histopathological sections were stained with Harris Hematoxylin and Eosin (H&E) stain for investigating the histopathological changes using a light microscope.

### Statistical analysis

All assays were estimated in triplicate, and the results were presented as means ± standard deviation (SD). Statistical analysis was performed using variance analysis one-way ANOVA via GraphPad Prism version 8.0.2, with significance determined at a *p-value* of less than 0.05.

## Result

### Bacterial strain characterization

**Fig. 1:**
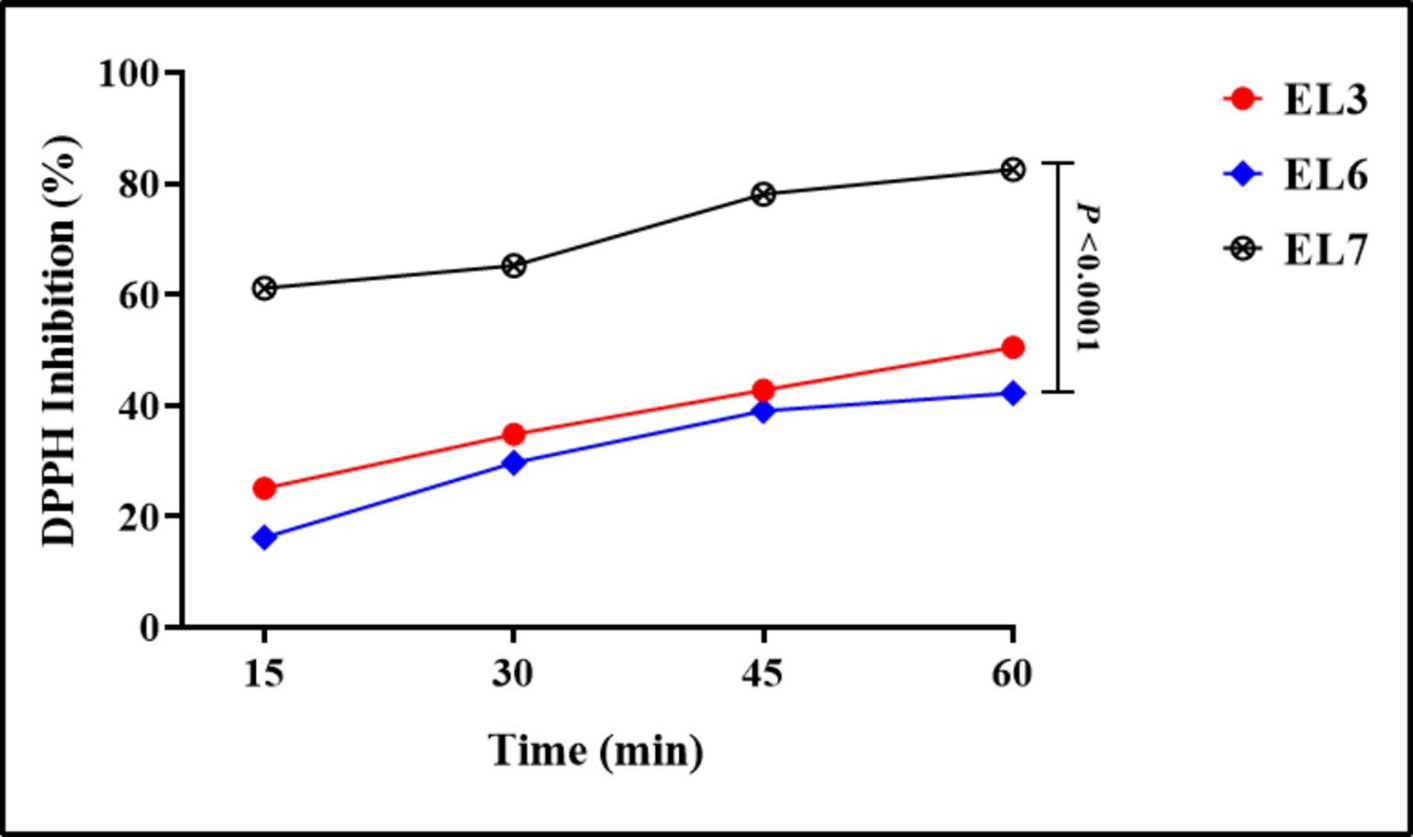
DPPH inhibition activity of the most three potent Exopolysaccharide isolated from (EL3, EL6, and EL7).

Seven bacterial isolates were recovered from the Red Sea (Sharm El Sheikh), Egypt. All isolates were subjected to the screening program to generate EPSs and antioxidant activity; the strains with significant antioxidant activity and EPS production were specified in Fig. 1. Three marine isolates (EL3, EL6, and EL7) produced a high quantity of EPS dry weight (8.3, 7.9, and 7.2 g/L) Table 1. These EPSs were screened against DPPH anti-oxidant activity and showed that EL7 was the most potent one (82.52 ± 0.7%). While, four isolates (EL1, EL2, EL4 and EL5) produced few amount of EPS with no antioxidant activity.

**Table 1:** Cell dry weight and EPSs productivity of the bacterial isolates.

Isolate No.	cell dry wt. (g/L)	EPS dry wt. (g/L)
EL1	4.1	2.9
EL2	2.7	3.4
EL3	4.3	8.3
EL4	3.2	5.2
EL5	4.4	2.6
EL6	4.5	7.9
EL7	4.2	7.2

**Fig. 2:**
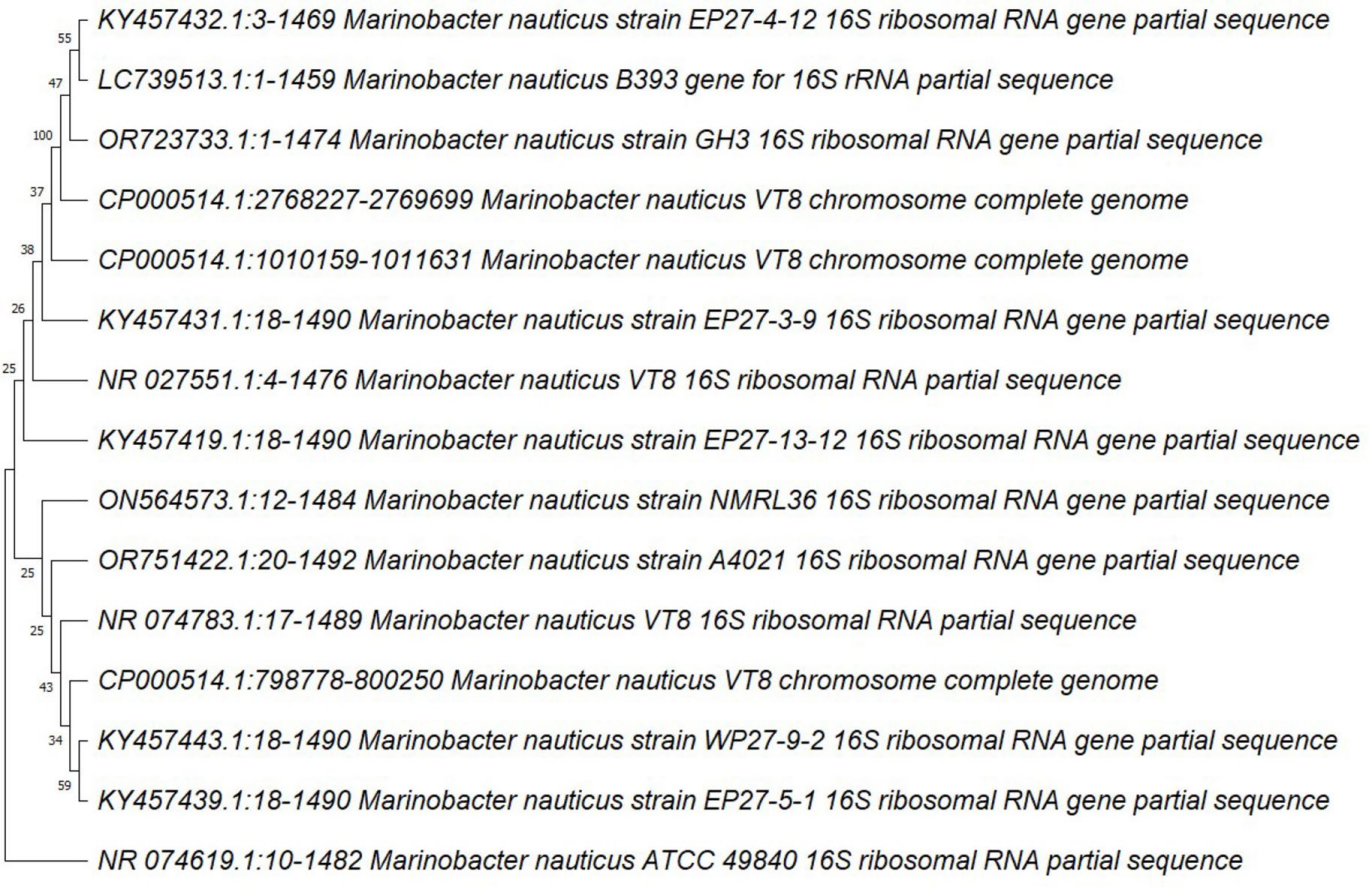
Phylogenetic tree of *Marinobacter nauticus* strain GH3 with accession number of (OR723733.1).

The most potent isolate (EL7) had a gram-negative, citrate-positive, and indole-negative response. The 16 S rRNA was sequenced after extracting the genomic DNA. The phylogenetic tree was constructed using the neighbor-joining technique. The genomic structure of the strain is most similar to the *Marinobacter nauticus* strain in the NCBI database, with a 100% match in terms of identity. Therefore, the bacterial strain that was separated was given the name *Marinobacter nauticus* GH3. The 16s rRNA gene of this strain was then submitted to the NCBI GenBank database with the assigned query ID (OR723733.1), as shown in Fig. 2.

### GH3-EPS purification and characterization

After the EPS production screening program, *Marinobacter nauticus* strain GH3 was cultivated in a medium that promotes the production of EPS. Exopolysaccharide was re-dissolved in deionized water and then dialyzed against deionized water for 72 h to undergo partial purification and fractionation. Four volumes of absolute cold ethanol were used to precipitate the dialyzed solution. The isolated EPS appears grayish-white in color, amorphous, and odorless. While it cannot dissolve in water, it does have the ability to dissolve in chloroform, ethanol, DMSO, and acetonitrile, among other organic solvents. The absence of protein and nucleic acid in the isolated EPS was confirmed by the biochemical analysis. Then it was purified with a DEAE-cellulose column, coded GH3-EPS.

**Fig. 3:**
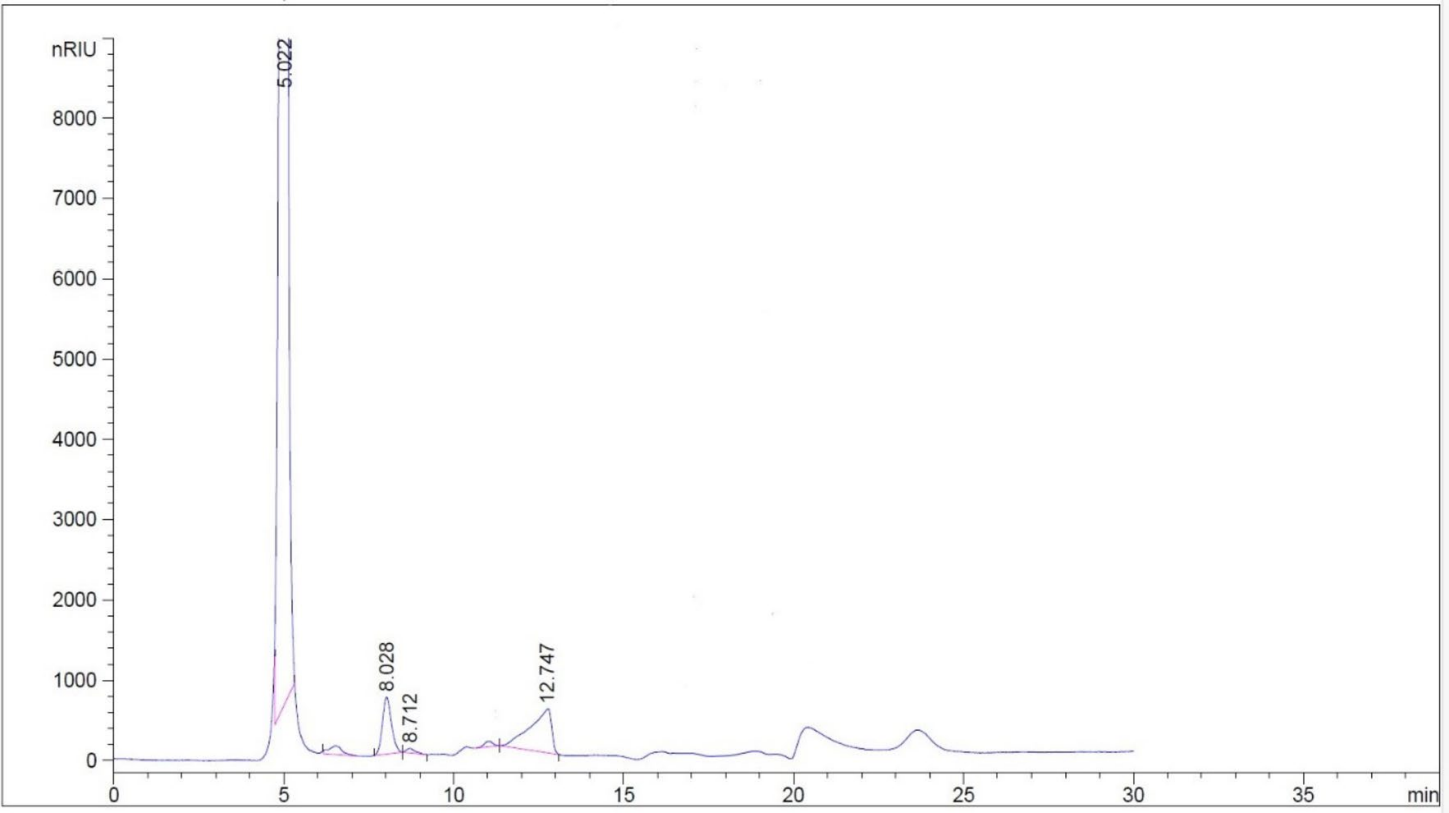
HPLC analysis of GH3-EPS for residues composition.

**Fig. 4:**
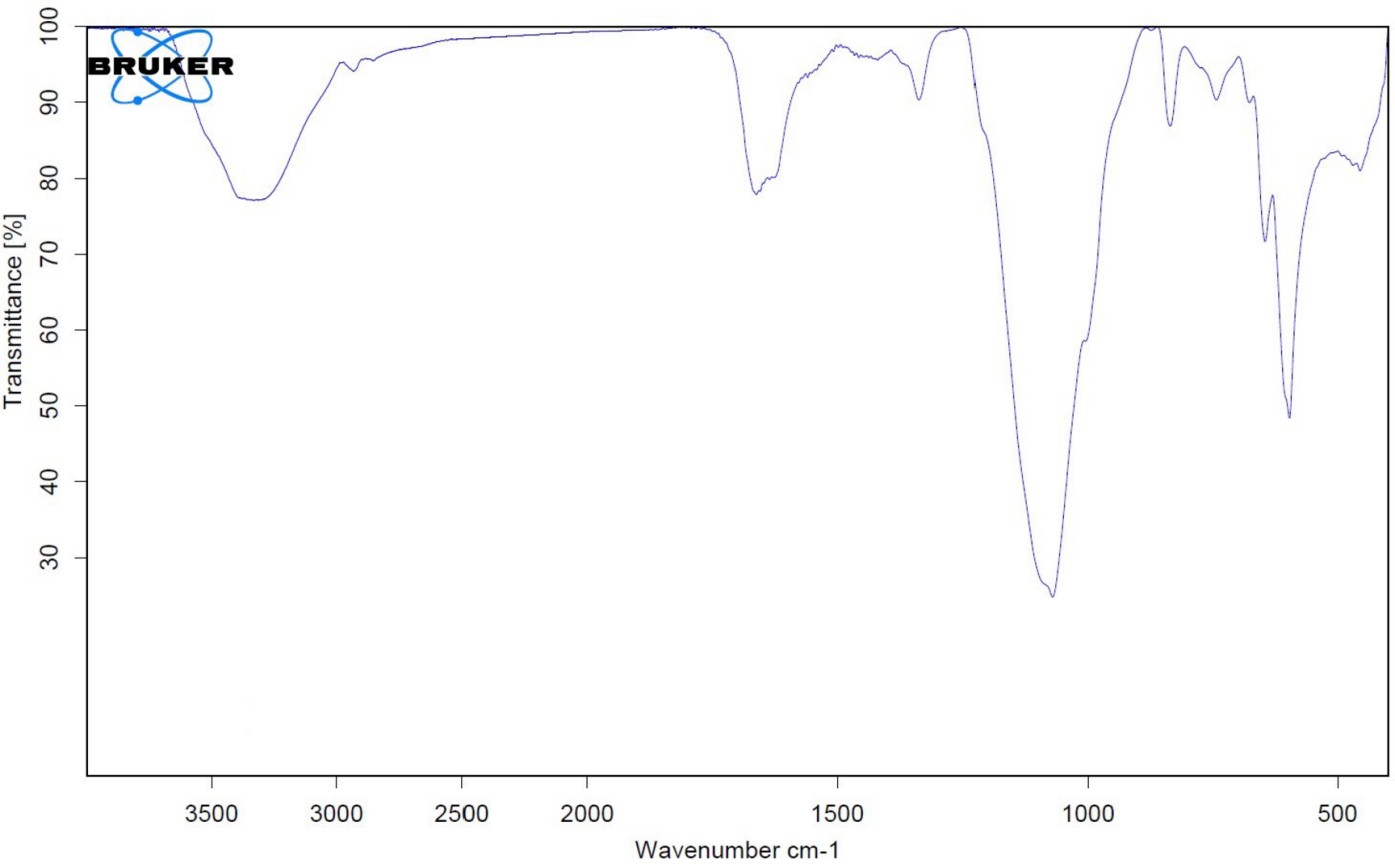
FTIR analysis of GH3-EPS showing the functional groups.

**Fig. 5:**
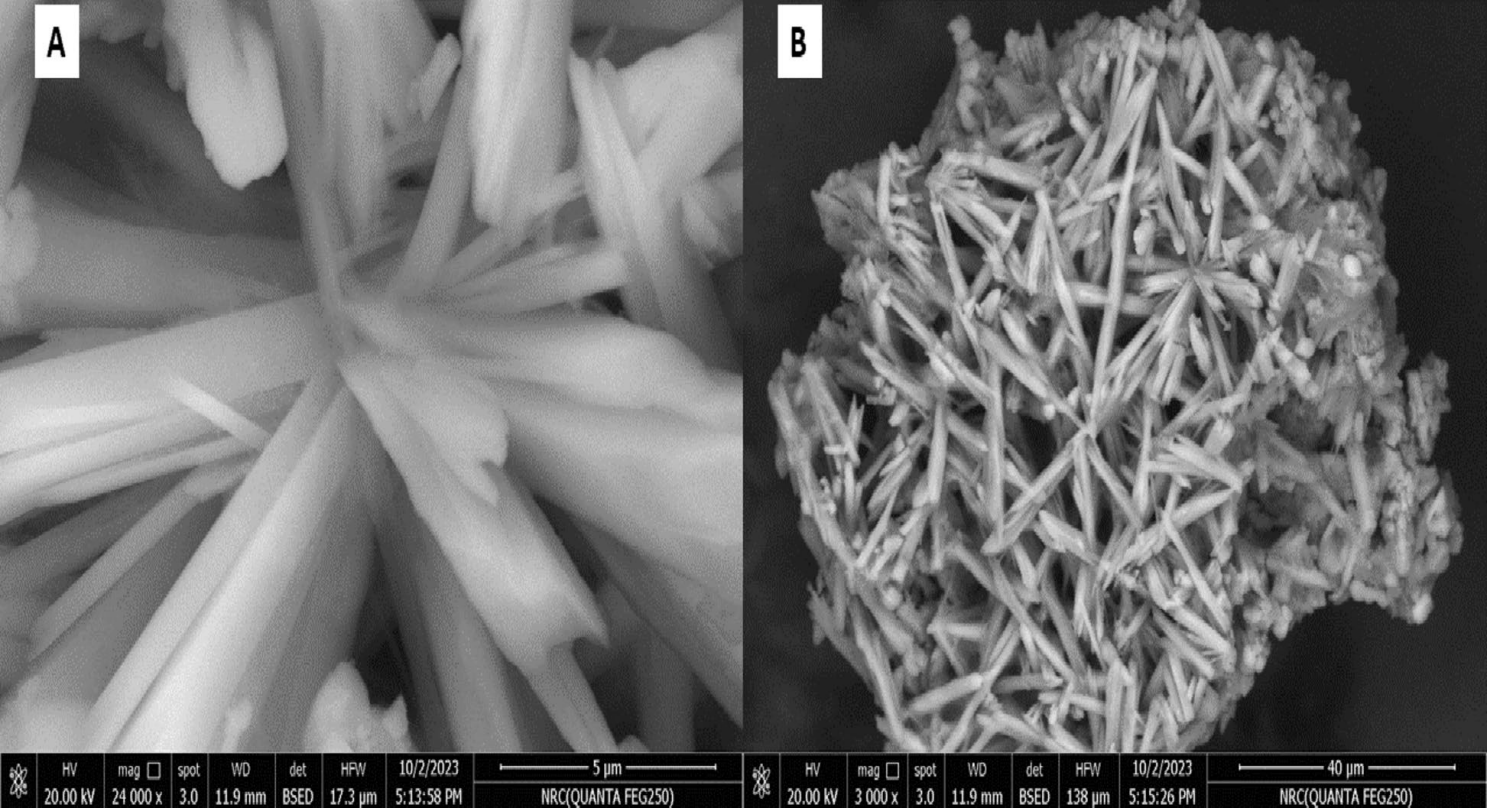
The surface morphology of GH3-EPS observed by SEM. (**A**) and (**B**); showed different features with different magnification at (24 000x and 3 000x mag.) respectively.

There was a sulfate content in the GH3-EPS at 25.4%, although it contains uronic acid and N-acetylglucosamine at 12.18% and 16.07%, respectively. The high-performance liquid chromatography (HPLC) analysis of the isolated *Marinobacter nauticus* strain GH3. It was discovered that the EPS core unit is composed of glucose, glucuronic acid, xylose, and arabinose (2:4:3:3), respectively, as shown in Fig. 3; therefore, the GH3-EPS is regarded as heteropolymeric. The functional group present in the EPS of *Marinobacter nauticus strain* GH3 was analyzed by FTIR spectroscopy. At 3303.21 cm1, the OH group’s considerable stretching vibration was detected. Both the C-H bond stretching peak at 2937.37 cm1 and the methyl or methylene group bending peak at 1336.62 cm1 were identified. The carboxyl group was identified as the cause of the peak at 1662 cm1. The intense peak at 1097.76 cm1 is associated with the vibration ring, which is combined with the vibrations of the glycosidic bond (C-O-C) and the stretching vibrations of the side groups (C-OH) and the sulfonyl groups. The peak at 835.42 cm1 and 835.99 cm1 in the two exopolysaccharides GH3-EPS revealed alpha-anomeric carbon, respectively. GH3-EPS was confirmed free from protein content by using a UV spectrophotometer (Fig. 4). SEM images provide a visual morphological image of GH3-EPS. At a magnification of 6000 x, the surface was observed as a thick, white, soft bundle interfering with each other’s, forming a cave. At 3000-x magnification, it appeared as a thick stick forming a flower-like shape, as shown in Fig. 5.

### In-vivo toxicity evaluation of GH3-EPS

#### Blood chemistry (hematology, kidney, and liver)

GH3-EPS administration exhibited no acute toxicity towards the experimental rats. No mortality occurred within 48 h in the treated groups. There were no significant behavioral differences; GH3-EPS administration did not show any immediate harmful effects on the rats used in the trial. In addition, there were no deaths within 48 h in either the control group or the treatment group. There were differences between the groups in the first 24 h. As shown in Table 2, the kidney function (creatinine and urea) levels for the group treated with crude GH3-EPS showed no significant difference from the negative control. ALT, AST, ALP, Alb, and total protein values for liver function in the GH3-EPS-treated group were insignificantly different from those of the control group. Moreover, all mean hematology parameter levels are within the normal range taken before (0 day) and after (14 days) administration of GH3-EPS.

**Table 2:** Biochemical analysis before and after treatment of GH3-EPS.

Section	Test	Before treatment	After 7 Days of treatment	After 14 Days of treatment	*P* value
Hematology	RBCs	5.5 ± 0.1	5.7 ± 0.1	6.1 ± 0.3	0.0011
	Hb	10.9 ± 0.1	10.9 ± 0.3	11.1 ± 0.5	0.5794
	HCT	32.5 ± 0.7	34.5 ± 0.7	34.9 ± 0.6	0.0002
	MCV	65.3 ± 0.6	64.0 ± 1.5	65.1 ± 0.4	0.1111
	MCH	21.1 ± 0.5	21.5 ± 0.7	22.0 ± 0.7	0.1255
	MCHC	34.4 ± 0.7	32.3 ± 1.3	35.0 ± 0.8	0.0022
	PLT	673.0 ± 14.8	648.8 ± 7.0	649.3 ± 7.6	0.0039
	WBCs	5.5 ± 0.1	5.4 ± 0.2	5.6 ± 0.2	0.2298
Kidney Function	Creatinine	0.4 ± 0.1	0.5 ± 0.1	0.5 ± 0.1	0.2298
	Urea	41.3 ± 0.9	41.4 ± 2.0	41.3 ± 1.4	0.9926
Liver Function	ALT	60.8 ± 1.5	49.1 ± 0.7	52.5 ± 6.7	0.0017
	AST	37.4 ± 1.4	35.6 ± 1.5	37.3 ± 1.7	0.1578
	ALP	32.8 ± 2.1	44.8 ± 2.4	42.7 ± 7.1	0.0026
	ALB	3.5 ± 0.1	3.5 ± 0.1	3.6 ± 0.3	0.6452
	T. Protein	0.4 ± 0.0	0.4 ± 0.0	0.4 ± 0.0	0.3966

#### Histopathological examination

**Fig. 6:**
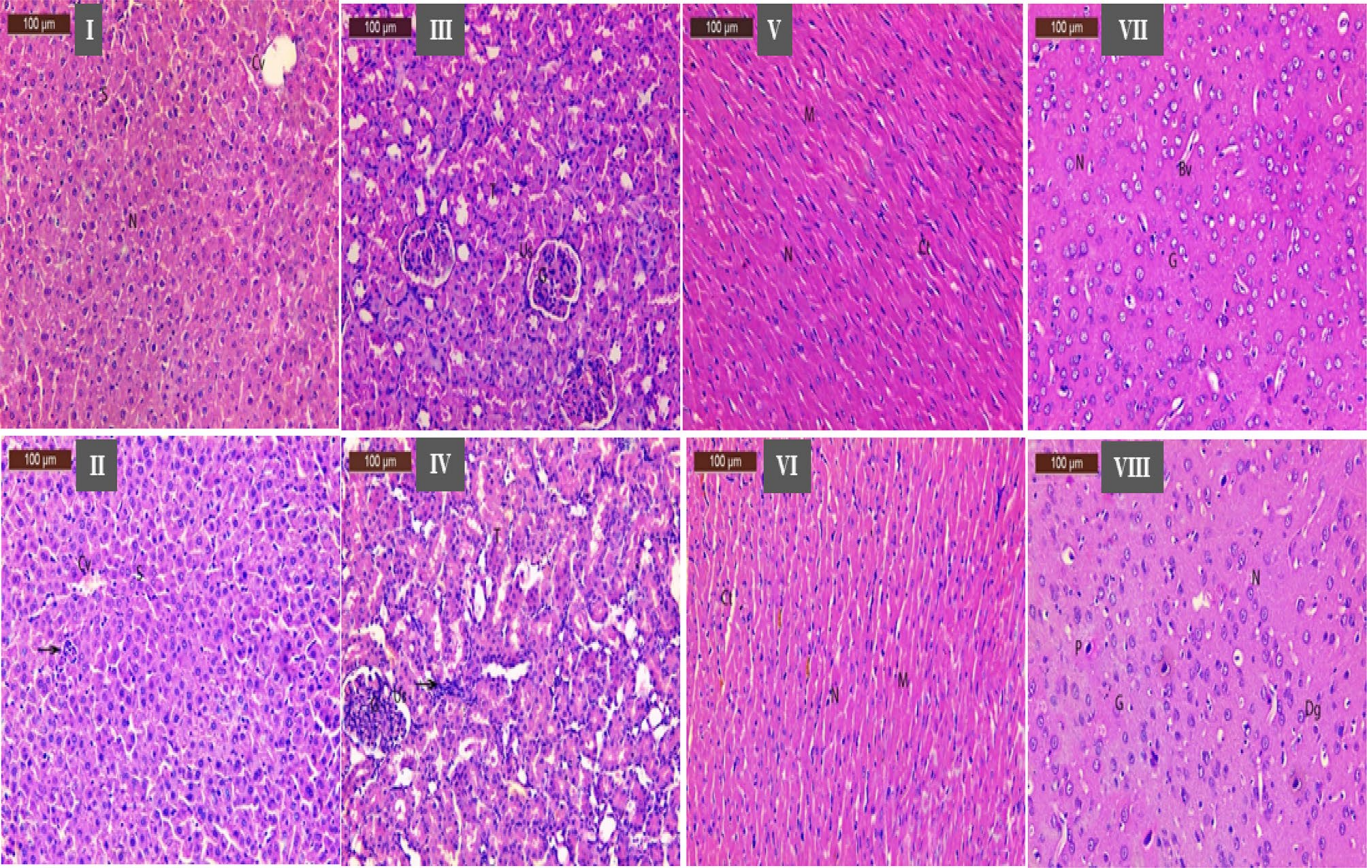
Histopathological toxicity study effect of GH3-EPS on some mice organs represented by photomicrographs as follow; control groups (I & III & V & VII) for (liver & kidney & heart & brain) respectively. And GH3-EPS treated groups as follow (II & IV & VI & VIII) for (liver & kidney & heart & brain) respectively.

Histopathological results were illustrated in Fig. 6. A photomicrograph of the liver control group showing normal hepatocytes (H) radiated from the central vein (CV) and separated by sinusoids (s) Fig. 6(I), and the liver treated group with GH3-EPS showed a nearly normal appearance of histological structure, normal of the central vein (Cv) with a slight dilated blood sinusoid (S) Fig. 6(II). For kidney, Photomicrograph of section of control group showing normal histological structure with normal appearance of renal corpuscle containing the glomerulus tuft (G) and surrounded by the urinary space (Us), intact renal tubules with vascular nucleus (T) Fig. 6(III), and the sections of the renal tissue group treated with GH3-EPS showed nearly normal histological structure of glomerulus (G), normal urinary space (Us), and renal tubules (T) appeared normal with few inflammatory cells (arrow) Fig. 6(IV). On the other hand, a photomicrograph of the heart section of the control group shows normal histological architecture of cardiac myocytes (M); most appear longitudinally with rounded vesicular centrally located nuclei (N); in-between the cardiac myocytes, there was a delicate layer of (CT) Fig. 6(V), and in the heart-treated group with GH3-EPS, it showed nearly normal histological architecture of cardiac myocytes (M), nuclei (N), and most myofibers were intact in the intercellular spaces, while a slight was dilated (Ct) Fig. 6(VI). In the brain section treated with GH3-EPS showed nearly normal histological architecture of structure brain tissue with normal neurons (N) and few appeared pyknotic (P), blood vessels with (Bv) and lightly (G) or dark stained nuclei glial cells (Dg), and few pyknotic nuclei (P) Fig. 6(VII), compared to the photomicrograph of the brain section of the control group, which showed normal histological architecture of structure brain tissue with normal neurons (N) blood vessels with (Bv) and lightly stained nuclei glial cells (G) Fig. 6(VIII).

### Immunomodulatory effects of GH3-EPS on Macrophage RAW264.7 cells

**Fig. 7:**
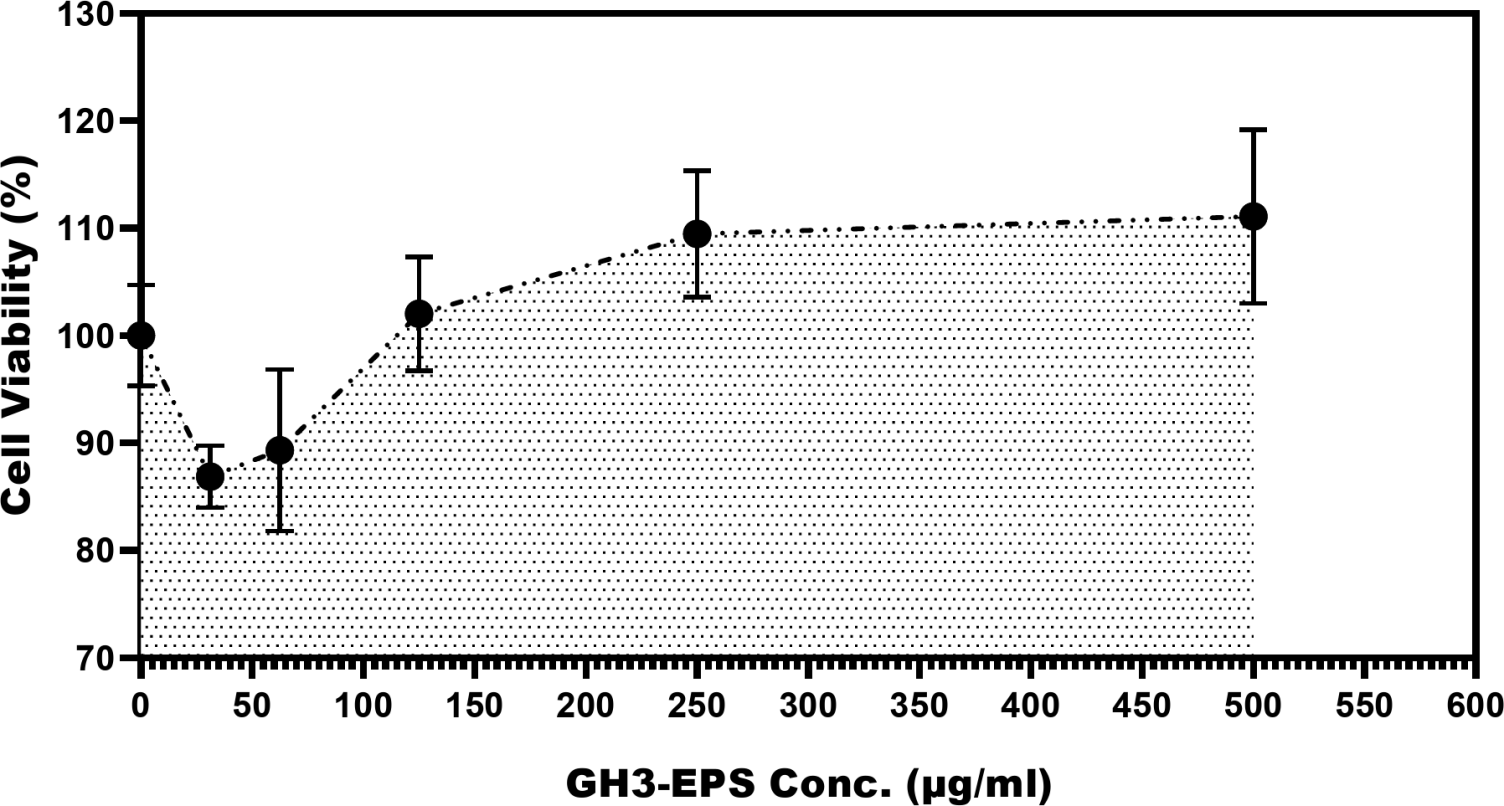
MTT assay for GH3-EPS cell viability estimation on macrophage RAW264.7 cells.

**Fig. 8:**
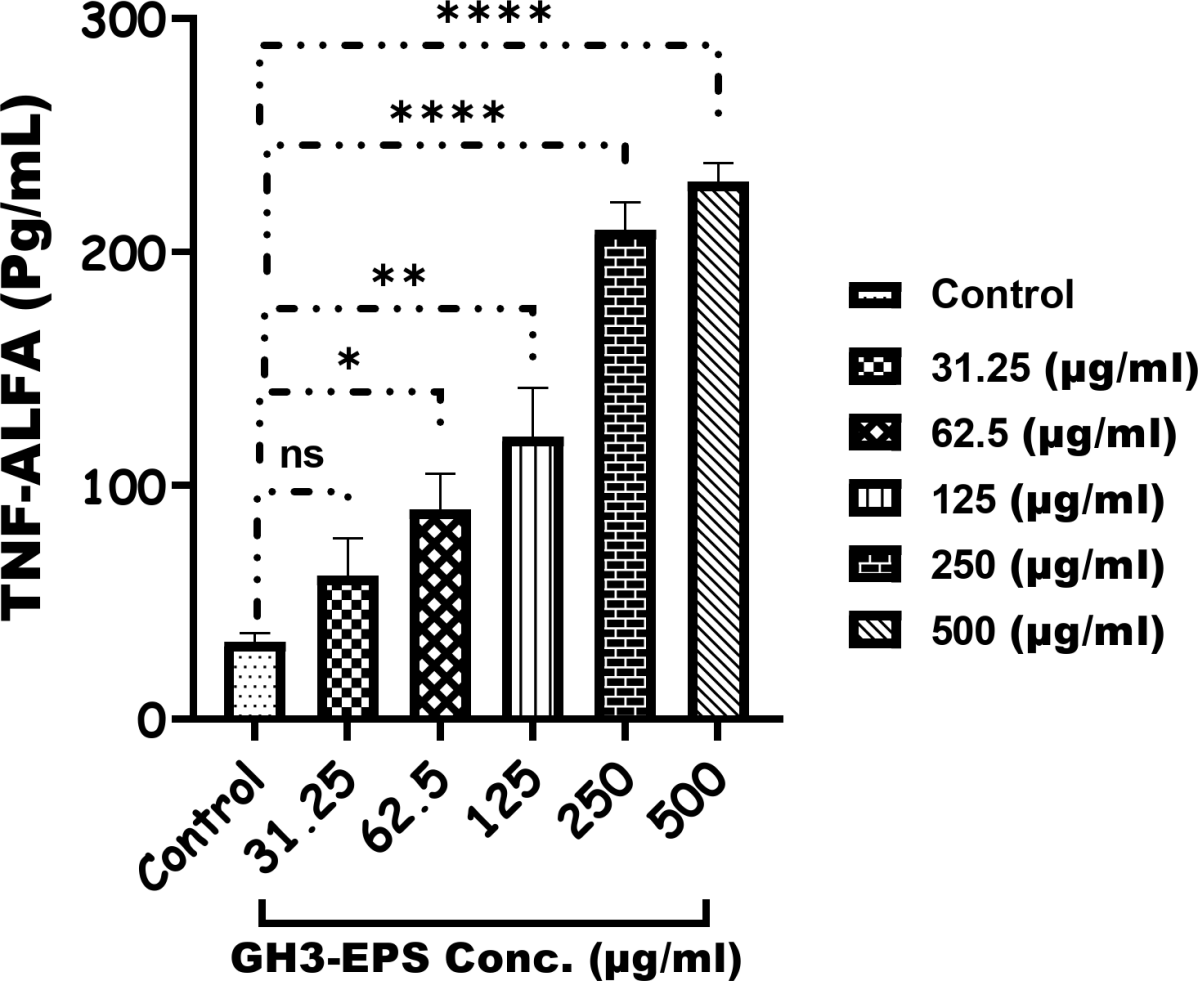
TNF-alfa production by macrophage RAW264.7 cells after treatment of GH3-EPS.

**Fig. 9:**
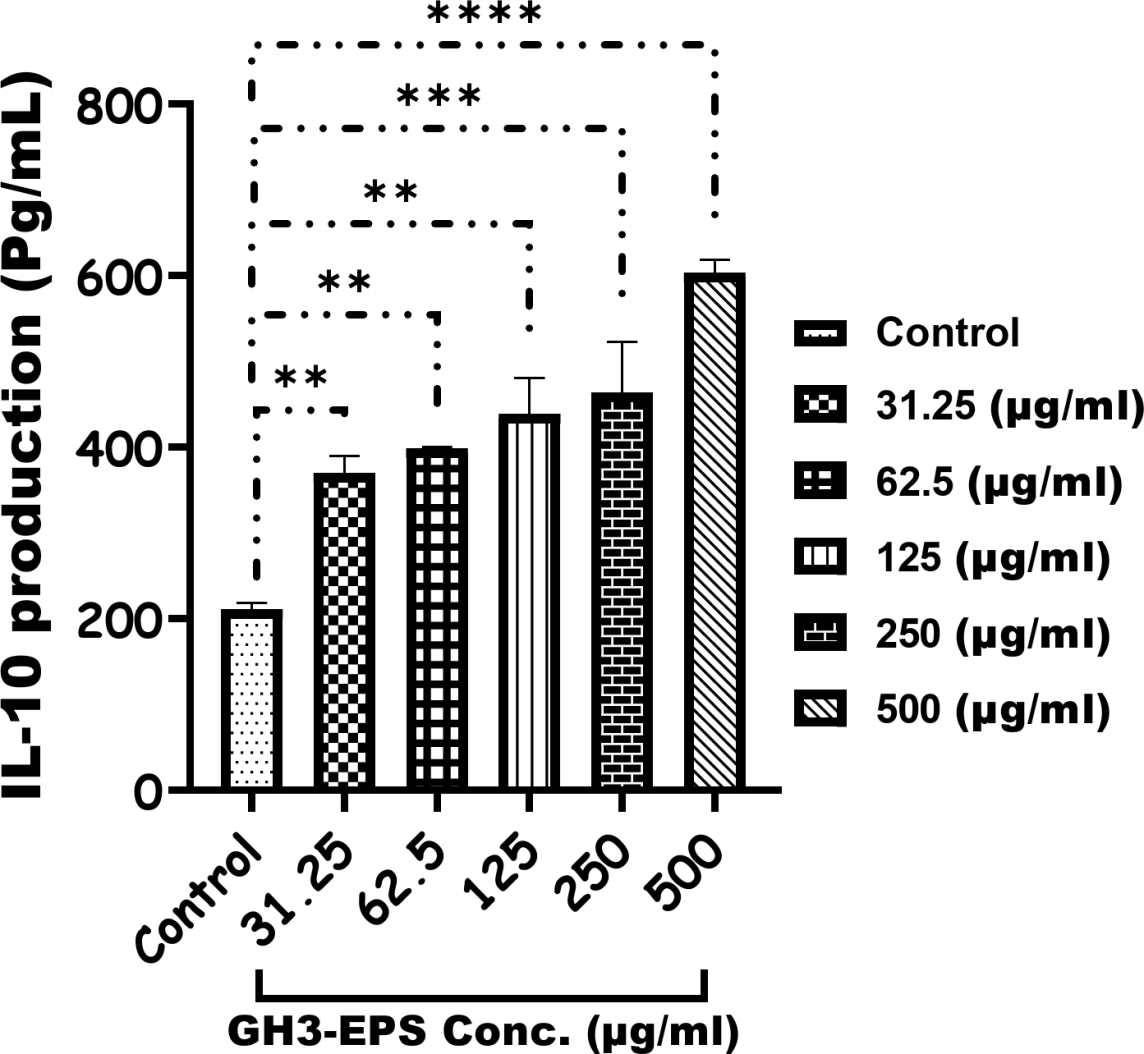
IL-10 production (Pg/ml) after treatment of GH3-EPS.

The cytotoxicity of GH3-EPS was evaluated by the cell vitality of macrophage RAW264.7 cells. As the results of the MTT assay. The vitality rate of RAW264.7 cells showed an increasing trend above 100%, which indicated that GH3-EPS in all concentration ranges used was not cytotoxic and could promote the proliferation of RAW264.7 macrophage cells. Figure 7. At all, when compared to the control group, GH3-EPS at concentrations of 125, 250, and 500 µg/mL considerably increased the vitality of RAW264.7 macrophages (*p* < 0.01). The data assessed using the respective ELISA kits indicated the expression of the immune cytokines (TNF-alpha and IL-10) rose in a manner that was dependent on the dosage (31.25–500 µg/mL) of treatment with GH3-EPS (Figs. 8 and 9).

### Antioxidant-related index assay

#### Enzymatic assay

**Fig. 10:**
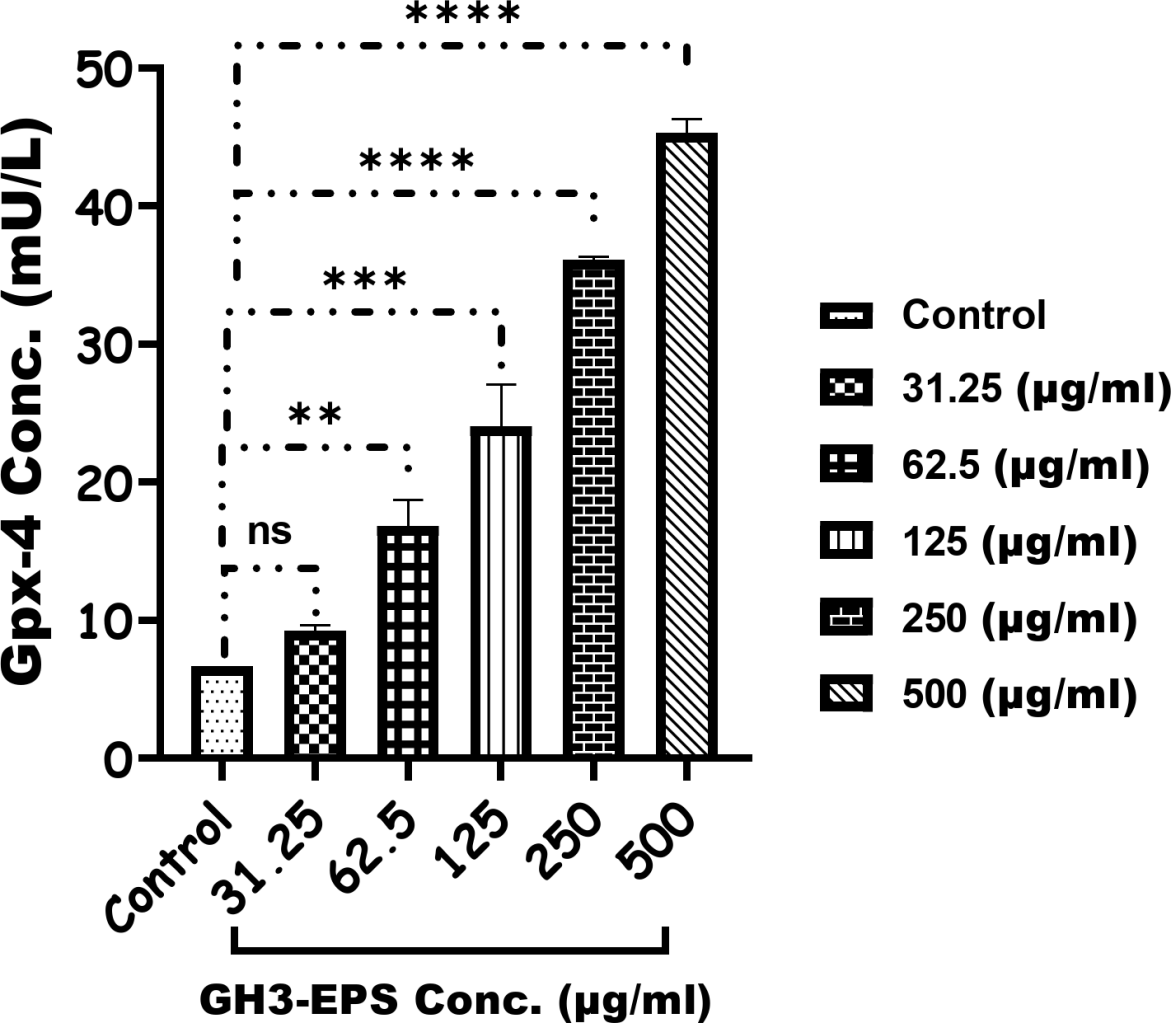
Glutathione peroxidase-4 (Gpx-4) concentration (mU/L) released after the treatment with GH3-EPS.

**Fig. 11:**
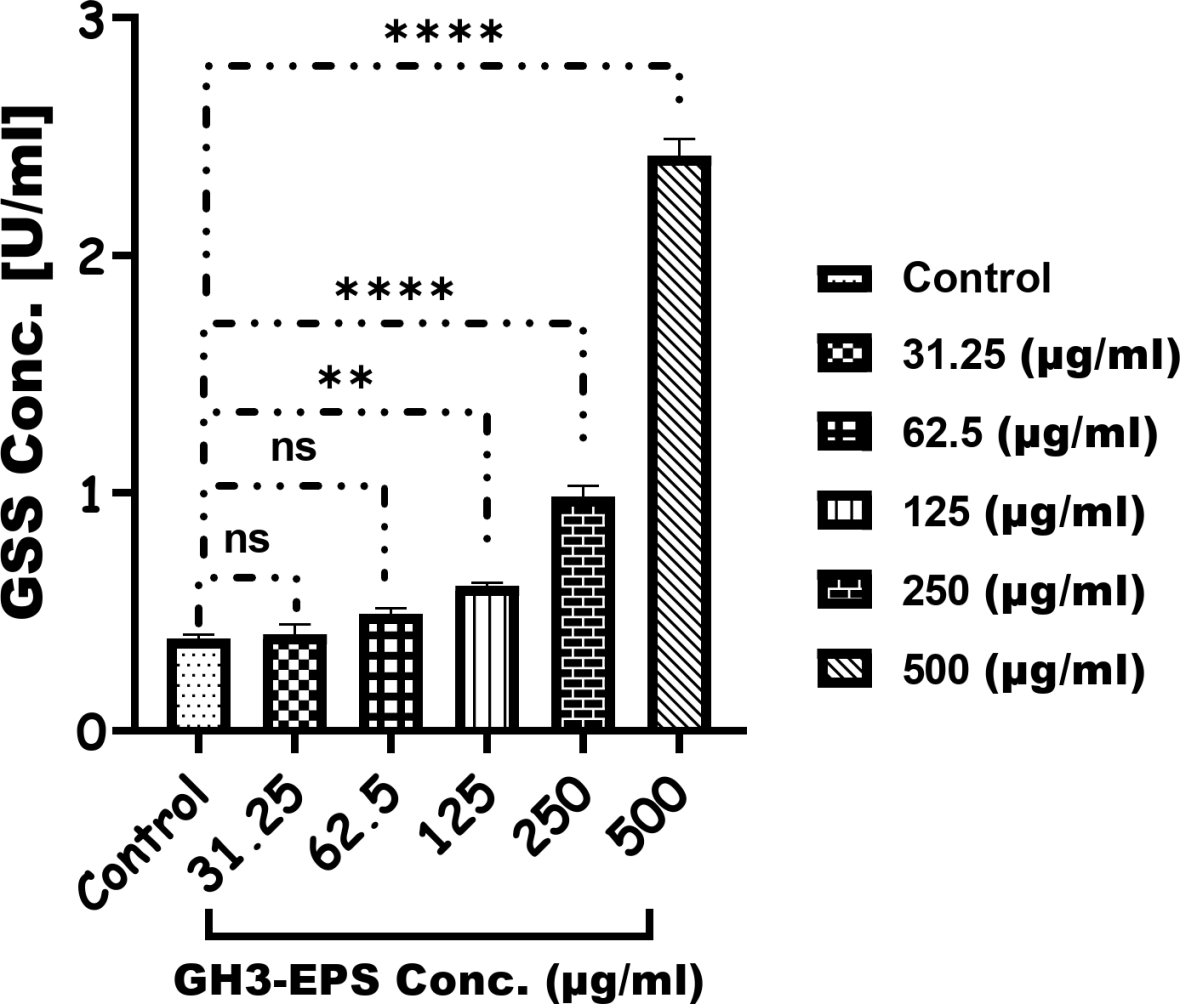
Glutathione synthetase (GSS) production with (U/ml) after the exposure to the GH3-EPS.

**Fig. 12:**
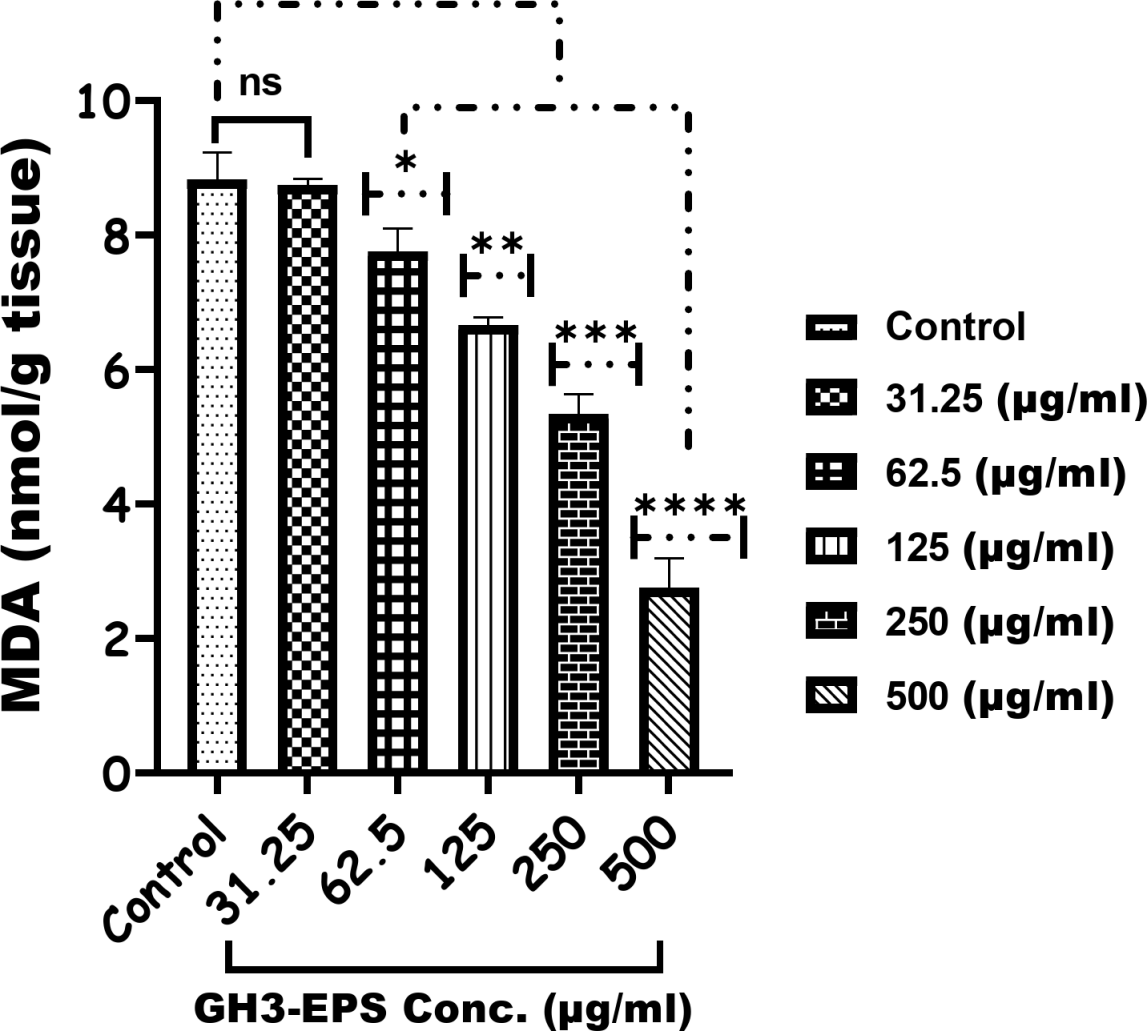
MDA by product of lipid peroxidation estimation after the treatment with GH3-EPS.

Figure 10 demonstrates a clear and direct correlation between the dosage and the activity of GPX-4 in the treatment groups compared to the control. The highest levels of activity were observed in the group treated with 500 µg/mL GH3-EPS, with a statistically significant difference (*P* < 0.0001). The GSS level in the treatment groups with concentrations of 31.25 and 62.5 µg/mL was not significantly different from the control group. However, the GSS level was highest in the treatment groups with concentrations of 125, 250, and 500 µg/mL of GH3-EPS, with a statistically significant difference (*P* < 0.0001) (Fig. 11). Conversely, the MDA level was significantly lower (2.755 ± 0.3 nmol/g tissue) in the GH3-EPS treatment, with the lowest activity recorded in the 500 µg/mL (*P* < 0.0001) Fig. 12, and there was no significant difference between the concentration of 31.25 µg/mL of GH3-EPS and the control group, giving a p-value of (*P* < 0.9982).

#### Chemical intervention ability of GH3-EPS (oxidative stress)

**Fig. 13:**
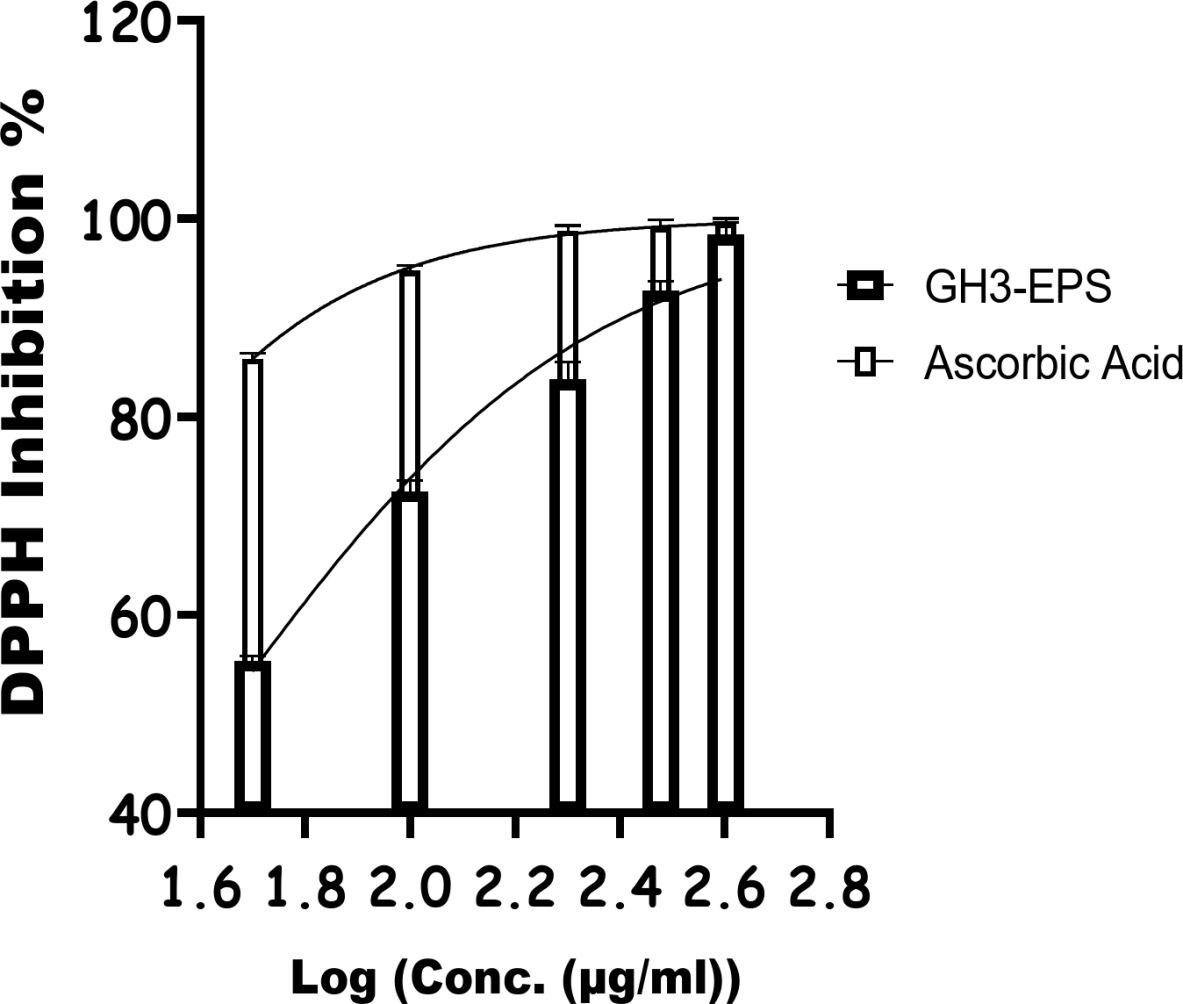
Antioxidant activity of GH3-EPS by DPPH assay.

**Fig. 14:**
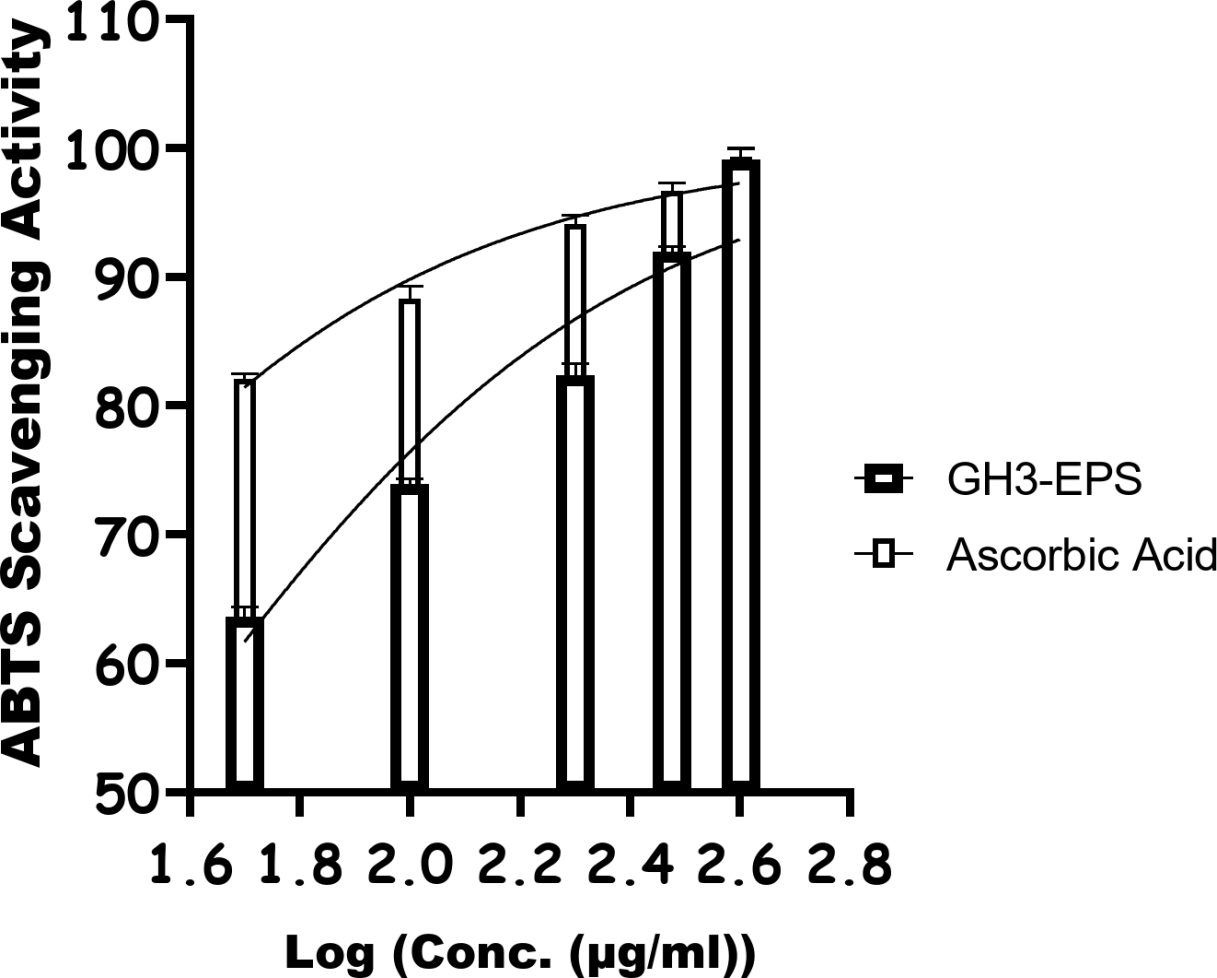
ABTS assay for GH3-EPS.

**Fig. 15:**
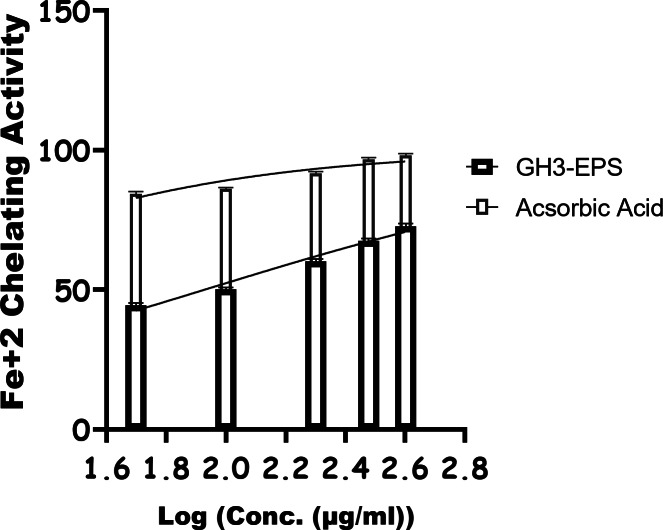
Iron chelating activity of GH3-EPS.

As shown in Fig. 13, GH3-EPS exhibited an obvious DPPH radical scavenging effect in a dose-dependent manner ranging from 50 to 400 µg/mL, enhancing its overall antioxidant activity. The scavenger efficiencies on DPPH radicals fell within a certain range (55.4% ± 0.5 to 98.4% ± 1.2) with an IC50 of 43.51 µg/mL, suggesting that GH3*-*EPS exerted extreme DPPH radical scavenging activity. As shown in Fig. 14, the ABTS detoxification capabilities of GH3-EPS exhibited a strong positive correlation with the concentrations, showing an increase while concentrations increased. The scavenging capacity of GH3-EPS on ABTS increased throughout the range of 63.58% ± 0.8 to 99.12% ± 0.8. The findings demonstrated that GH3-EPS had a significant ability to eliminate ABTS, as evidenced by its IC_50_ value of 31.27 µg/ml. Certain transition metals have the ability to stimulate the generation of harmful free radicals and intensify oxidative stress in cells. Fe, among the transition metals, exhibits high reactivity. Figure 15 demonstrates that the chelating capacities of GH3-EPS increased as their concentrations rose. The chelating activity on Fe^2+^ was 72.7% ± 1.0 at a concentration of 400 µg/mL with an IC_50_ of 84.96 µg/mL. These results revealed that our GH3*-*EPS demonstrated a strong ability to chelate Fe^2+^ ions.

### Inflammatory system intervention ability of GH3-EPS

**Fig. 16:**
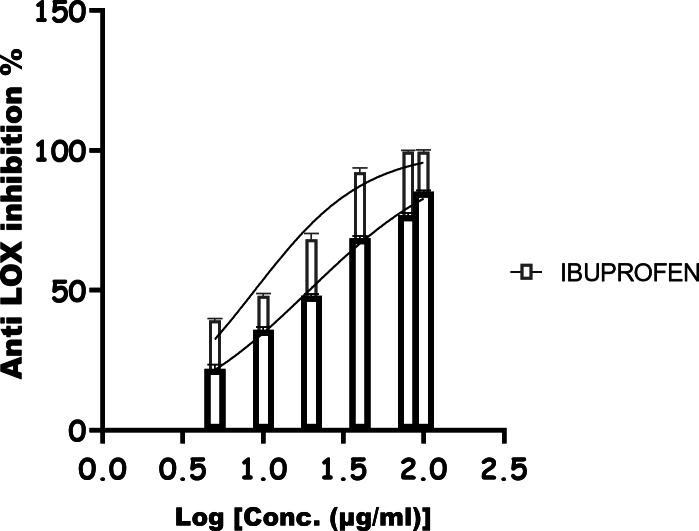
Anti-inflammatory effect of GH3-EPS against LOX.

**Fig. 17:**
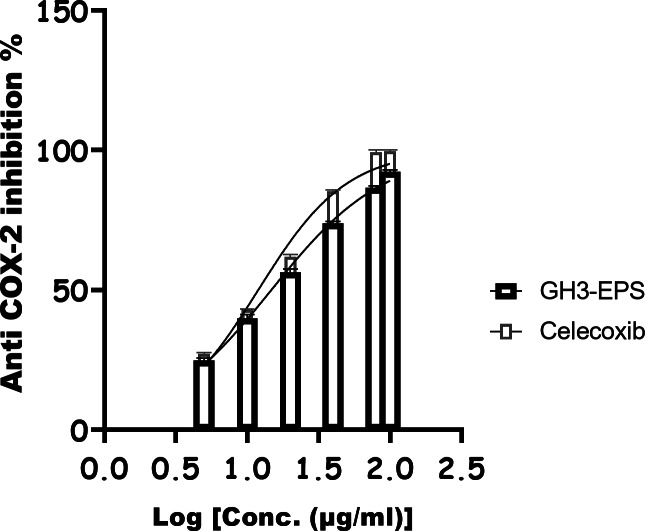
Anti-COX-2 effect of GH3-EPS as anti-inflammatory.

GH3-EPS anti-inflammatory activity was assessed using various methods, including the lipoxygenase (LOX) inhibitory as shown in Fig. 16, which had an IC_50_ of 19.40 µg/ml and the ibuprofen IC_50_ of 8.876 µg/ml. The COX-2 inhibitor shown in Fig. 17 gave 14.74 µg/ml, while the control (Celecoxib) gave 11.93 µg/ml.

### Cholinergic System Alternation ability of GH3-EPS

The AChE inhibitory activities of GH3-EPS were compared with those of donepezil. The GH3-EPS revealed a significant increase in anticholinesterase inhibitory activity; increasing the concentration of inhibitor (from 50 to 150 µg/ml) showed very effective AChE inhibitory activity (mean value: 52.92 ± 4.54 and 68.22 ± 5.64), respectively.

### In vivo findings of experimental fish

#### Observational and histopathological records for toxicity evaluation

**Fig. 18:**
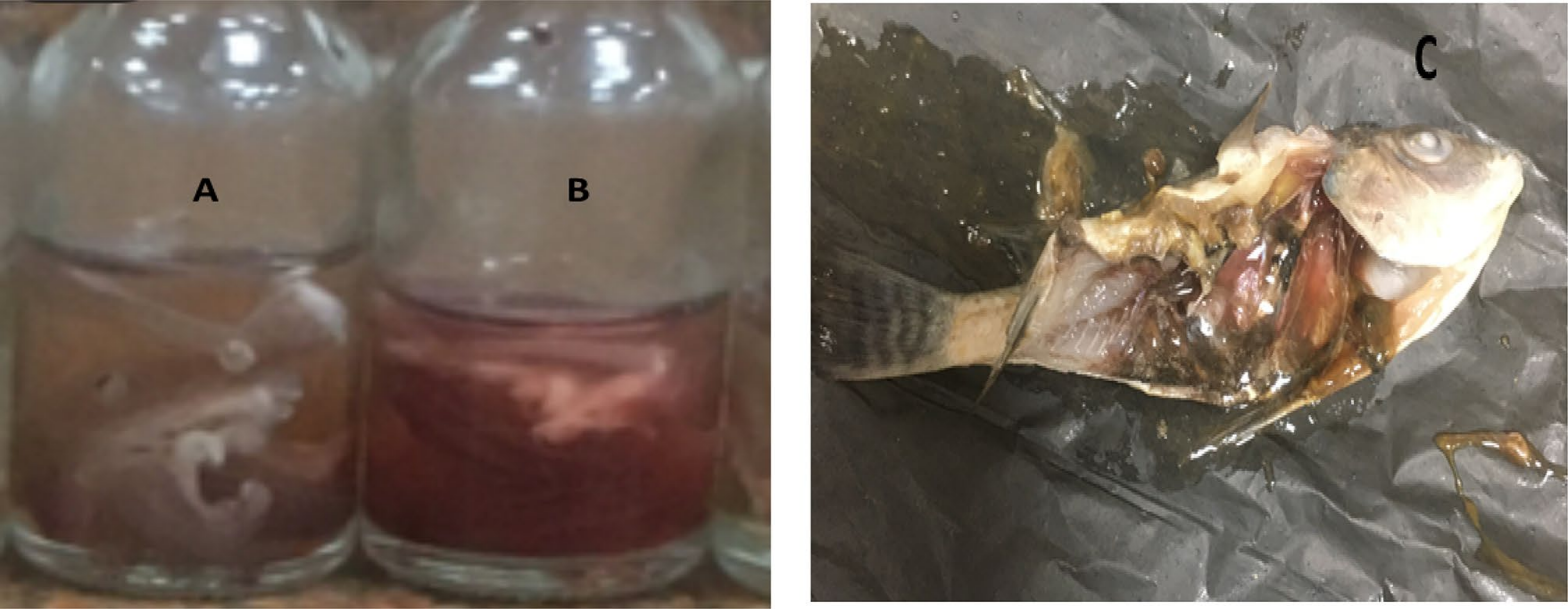
Observational result of the NiCl_2_ induced groups; (**A**) for fish gill color and texture; (C) for the digestive system. (**B**) illustrated the fish gill after the treatment of GH3-EPS.

We observed an increase in the numbers of dying fish in groups exposed to the toxicant NiCl_2_ positive control group after 25 days of exposure, and after examining the gills, discoloration, molting, and high softness, unlike usual “destroyed” gill filaments, were noticed Fig. 18(A). For treated groups with GH3-EPS, a fresh red color was observed Fig. 18(B). Also, damage was observed within the digestive system, especially the intestine tissue, which was totally damaged to varying degrees depending on time, with or without treatment with GH3-EPS in a dose-dependent manner Fig. 18(C).

**Fig. 19:**
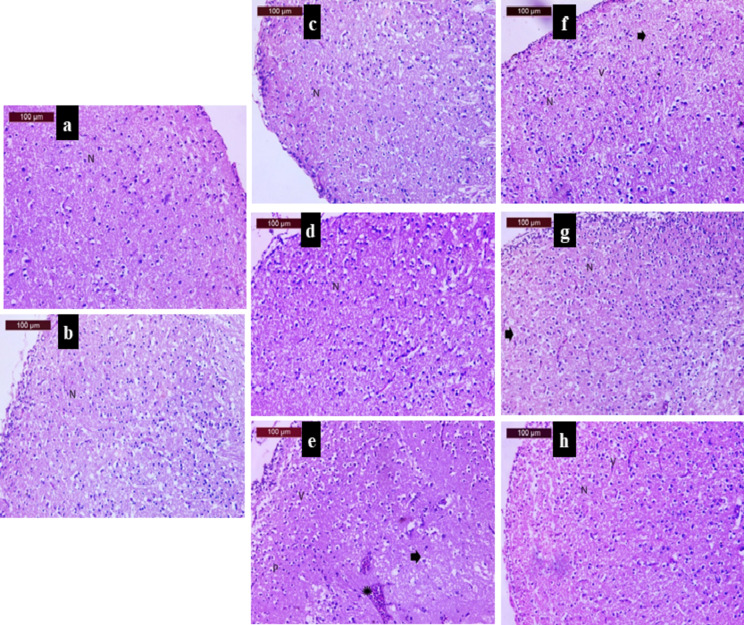
Histopathological study of GH3-EPS on fish after an exposure to NiCl_2_ was examined by photomicrographs of fish brain sections as follow; (**a**) Control group, (**b-d**) only GH3-EPS treatment groups in a dose dependent manner (Low, medium, and high), respectively. (**e**) NiCl_2_ induced group, and (**f-h**) NiCl_2_ induced groups with GH3-EPS treatment in a dose dependent manner (Low, medium, and high) respectively.

In the received GH3-EPS (low, medium, and high), toxicity assays are illustrated by photomicrographs of fish brain sections (Fig. 19). The fish brain control group Fig. 19 (a) showed normal architecture and neural cells with distinct nuclei (N). For the GH3-EPS low-dose treatment group Fig. 19(b), the medium-dose treatment group Fig. 19(c), and the high-dose treatment group Fig. 19(d), all showed nearly normal architecture and neural cells with distinct nuclei (N).

#### Histopathological investigation of GH3-EPS as an anti-AD

Histological examinations of fish brain sections after exposure to the LC_50_ of NiCl_2_ and the treatment of GH3-EPS with different concentrations (low, medium, and high) are illustrated in Fig. 19. The control group Fig. 19(a) showed normal architecture and neural cells with distinct nuclei (N) as described above. The NiCl2 group Fig. 19(e) showed degenerative changes (arrowhead), perivascular vacuolation (V), deeply stained pyknotic nuclei (P), and gliosis (star). On the other hand, the brain section of group NiCl_2_ treated with the low dose of GH3-EPS showed moderate improvement with nearly normal neuron (N), moderate degenerative changes (arrowhead), and slight perivascular vacuolation (V) Fig. 19(f). The brain section of group NiCl2, which was treated with a medium dose of GH3-EPS, showed oblivious improvement with nearly normal neurons (N); mild degenerative changes were observed in the neural cells (arrowhead) (Fig. 19g). The brain section group of NiCl_2_ treated with the high dose of GH3-EPS showed noticeable improvement with nearly normal neurons (N) and slight perivascular vacuolation (V) (Fig. 19h).

## Discussion

The exopolysaccharides derived from marine bacteria have significant uses in pharmaceuticals and medical treatments because of their effectiveness, safety, biocompatibility, and low immunogenicity. These EPS mostly find use in drug administration, adhesion, wound dressing, immunomodulatory, antibacterial, anti-inflammatory, anticoagulant, antiviral, and tissue engineering applications. This renders them highly appealing in comparison with polysaccharides derived from algae and plants. Maritime EPS are synthesized by diverse aquatic microbes, primarily belonging to the genus *Marinobacter* sp., among others^46,47^. Their versatility makes them useful in a wide range of contexts. The fields of composition and structure encompass various applications, including biosurfactants, medicine, and pharmaceuticals, with properties including those that regulate the immune system, fight cancer, reduce inflammation, prevent blood clots, and eliminate viruses. Biosurfactants are also used as additives in food and cosmetics, providing functions such as emulsifying, thickening, controlling viscosity, and gelling. Additionally, biosurfactants are employed in bioremediation processes, aiding in flocculation, absorption, oil recovery, and the removal of heavy metals, as well as serving as a source of sugar monomers for the production of biofuels and biochemicals. Additionally, they find applications in industries such as agriculture, detergent, paint, paper, and textiles^48,49^. Jiang et al.^50^. found that seawater samples collected from 22 stations in the photic zone of the Western North Pacific Ocean, ranging in depth from 5 to 200 m, contained the genome sequence of *Marinobacter nauticus* XTF-138.

In 2018, Boujida et al.^51^. discovered that *Marinobacter* sp. N4 and *Halomonas* sp. developed noteworthy extracellular polymeric substances (EPS) that created stable emulsions and exhibited strong antioxidant properties. Regarding the previous discussion, we isolated *Marinobacter nauticus* strain GH3 from the Red Sea, Sharm El Sheikh, Egypt, with an accession number of (OR723733.1). In this study, a good yield of EPS was achieved with dry weight (7.2 g/L). Rats were not affected by treatment-related toxicity or death when given NCDC 400 orally multiple times at different doses and for different lengths of time. When it came to their behavior, skin, fur, eyes, and gastrointestinal systems, not a single one of the mice showed any signs of clinical toxicity, All high elevations lie within the reported normal range for mice^52^. This study joins efforts to develop drugs that promote fewer side effects in Alzheimer’s disease patients, and our results show a promising anti-AD action of exopolysaccharides (EPS) produced by *M. nauticus* train GH3. Over all, the histopathological analysis indicates that the administration of our GH3-EPS does not result in significant histological alterations or acute toxicity in the liver, kidney, heart, or brain tissues of the experimental mice^53^. AChE is a potent amyloid-promoting factor as compared with other associated proteins. AChE inhibitors play a vital role in preventing the formation of toxic oligomeric forms of amyloid peptide^54^.

Certain EPS components have the ability to readily convert into ionic forms, which facilitates the EPS’s chelating properties towards cations, including toxic metals. The ability of EPS to chelate metallic elements essential for cell viability, such as zinc, cobalt, and iron, therefore binding and concentrating them. In addition to their ability to capture valuable metals, EPSs can also effectively bind and remove harmful metallic compounds, making them a valuable tool in mitigating environmental contamination^55,56^. The study found that the strain GH3 EPS of *Marinobacter nauticus* exhibited a pattern of substantial metal resistance that was comparable to patterns observed in Antarctic marine bacteria, as described in previous studies by Caruso et al.^57^. The enhanced strain tolerance in the presence of a carbohydrate substrate, which stimulates EPS production, suggests that under stressful conditions like metal contamination, the toxicity of these contaminants can be reduced by lowering their concentration in the surrounding environment through the development of agents that can chelate free ions^57^. Bouzaiene et al. explore the exopolysaccharide (EPS) from Lactobacillus plantarum C7, highlighting its antibacterial, antioxidant, prebiotic, and anti-enzymatic properties. FTIR analysis reveals key functional groups like hydroxyl and carboxyl that enhance its bioactivity. This EPS supports gut health and improves food stability, suggesting its potential as a natural alternative to synthetic additives in the food industry^58^. The prior results by Ozturk and Aslim^59^ provided support for these assumptions, as they describe the increased efficiency of bacterial EPS generation in the presence of metals. Multiple authors have extensively documented the ability of bacterial EPSs to chelate heavy metals. Gupta & Diwan^6^ extensively investigated various ways for sorption of heavy metals based on polymer characterization. The adsorption capabilities of *Marinobacter* isolates’ EPSs against lead and copper at neutral pH were previously documented by Bhaskar and Bhosle^60^. Liberti et al.^61^ found that numerous in vitro tests, including ABTS, DPPH, FRAP, and iron and copper chelating assays, were used to assess the antioxidant activity of s-EPS. S-EPSs were not able to scavenge the ABTS and DPPH radicals, whereas slight but significant activity was observed for the chelation of iron and for ferric ion reduction assays.

However, there is limited research on this topic for these genera. In agreement with that, our GH3-EPS have excellent antioxidant activity for DPPH, ABTS, and iron chelating with IC50s of 43.51, 31.27, and 84.96 µg/ml, respectively, and highly significant results (p-value < 0.05) for glutathione synthesize (GSS) and glutathione peroxidase-4 (Gpx-4), reducing the MDA byproduct. THP-1 cells treated with PPS showed a dose-dependent rise in the levels of various cytokines (IL-10, IL-6, IL-1β, and TNF-α) in comparison to the control group. Significantly, the impact on the release of cytokines by THP-1 cells was particularly noticeable when exposed to lower concentrations of PPS (5 µg/mL)^62^. Activated macrophages secrete cytokines, thus controlling the body’s homeostasis by inducing cell differentiation and proliferation. IL-1β, IL-6, IL-10, and TNF-α are important cytokines and play a significant role in regulating immune responses^63^.

Han et al. found that the exopolysaccharide F1, derived from Lacticaseibacillus rhamnosus B6, significantly boosted phagocytic activity and TNF-α expression in RAW 264.7 macrophages, showing dose-dependent immunomodulatory effects^64^. In addition to that, GH3-EPS could enhance the immunomodulatory effect by increasing the cell viability above 100% and enhance the release of the pro-inflammatory GH3-EPS shod anti-inflammatory effects (LOX and COX2) with IC50s of 19.4 and 14.74 µg/ml, respectively. Tsvetanova, highlights the medicinal potential of beneficial microorganisms, including bacteria, yeast, and fungi. These microbes produce metabolites that inhibit excessive cytokine production, showing promise in managing chronic inflammation and related disorders^65^. Chemical studies performed on the extracellular polymeric substances (EPSs) generated from *Marinobacter nauticus* strain GH3 revealed that they possess an exopolysaccharidic nature. Similarly, chemical studies conducted on the EPSs produced by *Marinobacter sp.* W1-16 under optimal conditions showed that they consist primarily of monosaccharides, specifically Glc, Man, Gal, GalN, GalA, and GlcA, while our strain *Marinobacter nauticus* GH3 has a monosaccharide composition (glucose, glucuronic acid, xylose, and arabinose) of 2:4:3:3, respectively, and has a sulfate content of 25.4%, uronic acid (12.18%), and N-acetylglucosamine (13.6%). The monosaccharide composition of bacterial EPS is highly variable, but in cold-adapted bacteria, it typically includes galactosamine and mannose^66^, along with the more common galactose and glucose^67^. This investigation discovered each of these residues within the isolated EPSs from the strain GH3 of *Marinobacter nauticus*. The roles and characteristics of bacterial EPSs can be better understood if their chemical composition is known. Extracted EPSs from cold-adapted bacteria contain sulphate, N-acetylglucosamine, and uronic acid, which is commonly acknowledged as a dependable indication of the ability of EPSs to chelate heavy metals^66^. Our EPS’s uronic acid content from *Marinobacter nauticus* strain GH3 was higher in level content (12.18%) than that reported for *Marinobacter sp*. strain W1-16 (7%) isolated from seawater in the Antarctic^57^. EPSs, despite sharing a fundamental chemical structure, are capable of undergoing or exhibiting structural changes. These factors encompass the length of the chain, the presence of combinations and configurations involving various functional groups, bonding, and substituents. These fluctuations are influenced by external factors, including temperature, carbon supply conditions, and pH^7^. EPSs can be attributed to specific molecular configurations. The data given here have significant medical value, particularly in light of the crucial role of EPS characteristics in the anti-Alzheimer’s effect while considering the potential consequences of certain EPS features.

Fish gills are exposed directly to aquatic media. Therefore, it is sensitive to any change in the water component. It is clear that exposure of various species of fish to heavy metals in the environment is associated with obvious structural damage to the gill epithelium^68^. The biochemical, physiological, and morphological reactions of gills to changes in the aquatic environment serve as excellent biomarkers for environmental biomonitoring. They enable the assessment of contaminant levels and the corresponding biomarker responses^69^. Hence, alterations in the gills serve as significant markers for assessing the impact of pollutants on these creatures, as they can reveal the activation of cellular defense mechanisms against the impacts and/or the reactive oxygen species (ROS) produced during detoxifying processes^70^. We observed an increase in fish mortality, morphological changes in our fish gills after exposure to the toxicant NiCl_2,_ with or without treatment with GH3-EPS, depending on the time and dose of treatment, and also damage to the digestive system, especially in the intestine. According to our study, there was a rise in the quantity of dying fish in the group subjected to a high concentration of toxins after 30 days of exposure. Upon inspecting the gill filaments, researchers noticed patchy discoloration, localized erosion, and punctuate damage in the gills, gill rakes, and operculum. Additional damage that may be detected includes eye, skin, and fin damage. The severity of these irritants fluctuates considering the length of time and levels of concentration of jellyfish contact^71^.

The field of study known as histopathology involves using a microscope to analyze diseased or injured tissue. Effective disease diagnosis frequently depends on the histological examination of tissue samples, making it an essential tool in anatomical pathology. Multiple studies have discovered that various environmental pressures can result in distinct tissue damage within the fish brains^72,73^.

The histopathology analysis for the organs (gills, brain, and liver) of the fish was subjected to acute toxicity studies. Fish from four different groups (control, 50 mg, 100 mg, and 200 mg) were randomly selected. After the dissection of the fish, the major organ brain was collected and subjected to the histopathological analysis. G1: brain control; G2: brain treated with 50 mg/kg of concentration of sample; G3: brain treated with 100 mg/kg of concentration of sample; and G4: brain treated with 200 mg/kg of concentration of sample. The groups 1, 2, 3, and 4 were suggesting that the sample doesn’t affect the brain from the experimental period. The results obtained from this study clearly indicate that the brown seaweed Sargassum wightii crude aqueous extract was suggested to have an excellent antioxidant activity^74^.

The brain serves as the central control center for all bodily functions and motions in creatures, such as fish, acting as a relay station. The study by Chamarthi et al.^72^ found histological abnormalities in the brain of the fish *Cyprinus carpio* exposed to a sublethal dosage of quinalphos toxicity, including hyperplasia, edema, necrosis, and an increase in brain cells.

Multiple authors have documented various histological changes in the brains of fish following exposure to different chemical compounds^75,76^. In a study conducted by Das & Mukherjee^77^, it was found that the brains of Indian major carp (Labeo rohita) exposed to 0.35 ppm hexachlorocyclohexane showed mild vacuolar changes with empty spaces, but at 1.73 ppm, the researchers found severe necrosis of neuronal cells in the brain as well as loss of Nissl substance. In another way, the brain of the fish *Clarias gariepinus* was shown to have mononuclear infiltration, neuronal degeneration, and severe spongiosis after being exposed to toxic levels of cypermethin^78^. The fish *Oreochromis niloticus*, when subjected to lethal quantities of glyphosate, experienced severe mononuclear infiltration, hemorrhage, generalized spongiosis, and congestion in their brains^79^. The brain of the fish in the experiment exhibited disintegration and significant damage to the brain cells, as well as breakdown of neuronal bundles, following exposure to various concentrations of malathion^80^. In this study, GH3-EPS could alter the damage to the brain in relation to its different concentrations (low, medium, and high).

## Conclusion

EPS-producing strain, “*Marinobacter nauticus* GH3,” has been successfully isolated from Sharm El-Sheikh, Egypt. GH3-EPS is a heteropolysaccharide consisting of glucose, XXXlucuronic acid, xylose, and arabinose and consists of sulfate, uronic acid, and N-acetylglucosamine. GH3-EPS enhanced cell proliferation in RAW264.7 macrophages and showed high immunomodulatory effects on TNF-alpha and IL-10. It also enhanced the activity of GPx-4 and GSS with antioxidant activity against MDA, DPPH, ABTS, and iron chelating. It showed an anti-inflammatory effect against LOX and COX-2. Histopathological studies confirmed the non-toxic effect of GH3-EPS on mouse tissue organs (liver, kidney, heart, and brain) and on fish brain sections. It demonstrated a good treatment effect for Alzheimer’s disease in a fish animal model. The study clearly demonstrates that GH3-EPS has the potential to be considered as a viable option for future advanced medical research as a novel organic substance for developing medications to treat Alzheimer’s disease.

## Data Availability

All data generated or analyzed during this study are included in this published article.
